# BEENE: deep learning-based nonlinear embedding improves batch effect estimation

**DOI:** 10.1093/bioinformatics/btad479

**Published:** 2023-08-10

**Authors:** Md Ashiqur Rahman, Abdullah Aman Tutul, Mahfuza Sharmin, Md Shamsuzzoha Bayzid

**Affiliations:** Department of Computer Science and Engineering, Bangladesh University of Engineering and Technology, Dhaka 1205, Bangladesh; Department of Computer Science, Purdue University, West Lafayette, IN 47907, United States; Department of Computer Science and Engineering, Bangladesh University of Engineering and Technology, Dhaka 1205, Bangladesh; Department of Computer Science and Engineering, Texas A&M University, College Station, TX 77843, United States; Department of Genetics, Stanford University, Stanford, CA 94305, United States; Department of Computer Science and Engineering, Bangladesh University of Engineering and Technology, Dhaka 1205, Bangladesh

## Abstract

**Motivation:**

Analyzing large-scale single-cell transcriptomic datasets generated using different technologies is challenging due to the presence of batch-specific systematic variations known as batch effects. Since biological and technological differences are often interspersed, detecting and accounting for batch effects in RNA-seq datasets are critical for effective data integration and interpretation. Low-dimensional embeddings, such as principal component analysis (PCA) are widely used in visual inspection and estimation of batch effects. Linear dimensionality reduction methods like PCA are effective in assessing the presence of batch effects, especially when batch effects exhibit linear patterns. However, batch effects are inherently complex and existing linear dimensionality reduction methods could be inadequate and imprecise in the presence of sophisticated nonlinear batch effects.

**Results:**

We present **B**atch **E**ffect **E**stimation using **N**onlinear **E**mbedding (BEENE), a deep nonlinear auto-encoder network which is specially tailored to generate an alternative lower dimensional embedding suitable for both linear and nonlinear batch effects. BEENE simultaneously learns the batch and biological variables from RNA-seq data, resulting in an embedding that is more robust and sensitive than PCA embedding in terms of detecting and quantifying batch effects. BEENE was assessed on a collection of carefully controlled simulated datasets as well as biological datasets, including two technical replicates of mouse embryogenesis cells, peripheral blood mononuclear cells from three largely different experiments and five studies of pancreatic islet cells.

**Availability and implementation:**

BEENE is freely available as an open source project at https://github.com/ashiq24/BEENE.

## 1 Introduction

Recent technological advancements have paved the way to generate large-scale single-cell RNA sequencing (scRNA-seq) data, leading to the establishment of large-scale projects such as the Human Cell Atlas (Regev *et al.* 2017). Due to logistical constraints in such large studies, scRNA-seq data is often generated and compiled from multiple experiments with differences in laboratory conditions, reagent lots, capturing times, and personnel differences (Leek *et al.* 2010, Haghverdi *et al.* 2018, Tran *et al.* 2020). As such, integrative analysis of several scRNA-seq datasets from different cohorts and studies is inevitable to acquire comprehensive and meaningful biological information (Rhodes and Chinnaiyan 2005, Korsunsky *et al.* 2019). However, meaningful biological variations are often confounded by the unwanted technical variations introduced by nonbiological factors of the experiments (Luo *et al.* 2010). This qualitatively different behavior across conditions that are unrelated to the biological or scientific variables is known as batch effects.

Batch effects are widespread and critical to address. As such, identification and removal of batch effects while preserving the biological variations have received considerable attention from the scientific community. ComBat (Johnson *et al.* 2007), CombBat-Seq (Zhang *et al.* 2020), limma (Ritchie *et al.* 2015), RUVseq (Risso *et al.* 2014), svaseq (Leek 2014), Seurat-CCA (Butler *et al.* 2018), MNN Correct (Haghverdi *et al.* 2018), BERMUDA (Wang *et al.* 2019), DESC (Li *et al.* 2020), and Harmony(Korsunsky *et al.* 2019) are among the well-known state-of-the-art batch correction methods. In addition to these batch correction techniques, methods for *batch effect assessment* are also of high interest to effectively detect and measure batch effects. The primary approach for batch effect detection involves visual inspection of low dimensional representation of data, such as PCA, t-SNE (Van der Maaten and Hinton 2008), and UMAP (McInnes *et al.* 2018). Batch evaluation methods, involving interactive visualizations and statistical analyses to evaluate batch effects and explore their impact on the data, have also been developed (Manimaran *et al.* 2016). However, visual inspections are subjective and do not provide any quantitative measurements to assess and compare the level of batch effects present in the data. As a result, several quantitative measures for assessing batch effects have been developed. Average Silhouette Width (Rousseeuw 1987, Lotfollahi *et al.* 2018) assesses batch effects by silhouette scores, which represents the similarity of data points in its own cluster compared to other clusters. The Adjusted Rand Index (Hubert and Arabie 1985), which computes the similarity between two clusters, can also be used for batch effect assessments. *k*-nearest neighbor batch-effect test (kBET) (Büttner *et al.* 2019) and Local Inverse Simpson’s Index (LISI) (Korsunsky *et al.* 2019) are two well-known “local” batch effect measurement metrics. The kBET metric measures batch mixing by considering the deviation between local and global distributions using a predetermined number of nearest neighbors. LISI measures the local batch mixing “integration LISI” (iLISI) by computing the inverse Simpson’s index on the local neighborhood based on a predefined perplexity. These assessment metrics mostly involve calculating distances between data points in lower dimensional embeddings generated by linear dimensionality reduction methods such as PCA.

Batch effects, on the other hand, are complex in nature and can be highly nonlinear (Tran *et al.* 2020), making it difficult for the embeddings generated by linear dimensionality reduction methods like PCA to correctly detect and assess the degree of batch effects present in the data. The application of nonlinear transformations to learn nonlinear mappings from the data domain to low-dimensional latent space may lead to improved clustering of data (Salakhutdinov and Hinton 2007, Ren *et al.* 2014, Yang *et al.* 2017). However, nonlinear transformations that are specially tailored to generate embeddings capable of capturing both biological and batch variability in the face of both linear and nonlinear batch effects are currently lacking.

Here, we present **B**atch **E**ffect **E**stimation using **N**onlinear **E**mbedding (BEENE) to correctly estimate linear as well as complex nonlinear batch effects in RNA-seq data. The advantage of nonlienar transformations, as used in BEENE, is its ability in modeling the higher-order correlations among the original data dimensions (Salakhutdinov and Hinton 2007). BEENE exploits the ability of a deep autoencoder to approximate nonlinear mappings, which is guided by two separate neural networks to capture batch-specific and biological variability in the data. Thus, BEENE embeds cells into a lower-dimensional space using nonlinear transformation, which can explain batch variability while retaining biological variables in the presence of both linear and complex nonlinear batch effects. As we will show in the following that the BEENE embeddings are, in fact, more robust and sensitive to the presence of batch effects than PCA regardless of whether the batch effects are linear or nonlinear. The performance of BEENE was assessed on a collection of simulated datasets covering a wide range of model conditions with both linear and nonlinear batch effects, as well as several single-cell RNA-seq datasets, including two technical replicates of mouse embryonic stem cells (mESCs), peripheral blood mononuclear cells (PBMC) from three different experiments, and five studies of pancreatic islet cells. The performance of BEENE was evaluated in comparison to both traditional PCA and existing batch assessment methods such as kBET and LISI. Furthermore, we evaluate its performance before and after batch correction using widely used batch correction approaches, namely ComBat (Johnson *et al.* 2007), ComBat-Seq (Zhang *et al.* 2020), MNN Correct (Haghverdi *et al.* 2018), and Harmony (Korsunsky *et al.* 2019).

## 2 Materials and methods

We start with an overview of the overall pipeline used in BEENE in Section 2.1 followed by the detailed descriptions of the architecture of BEENE in the subsequent section.

### 2.1 Overview of BEENE

**Figure 1.**
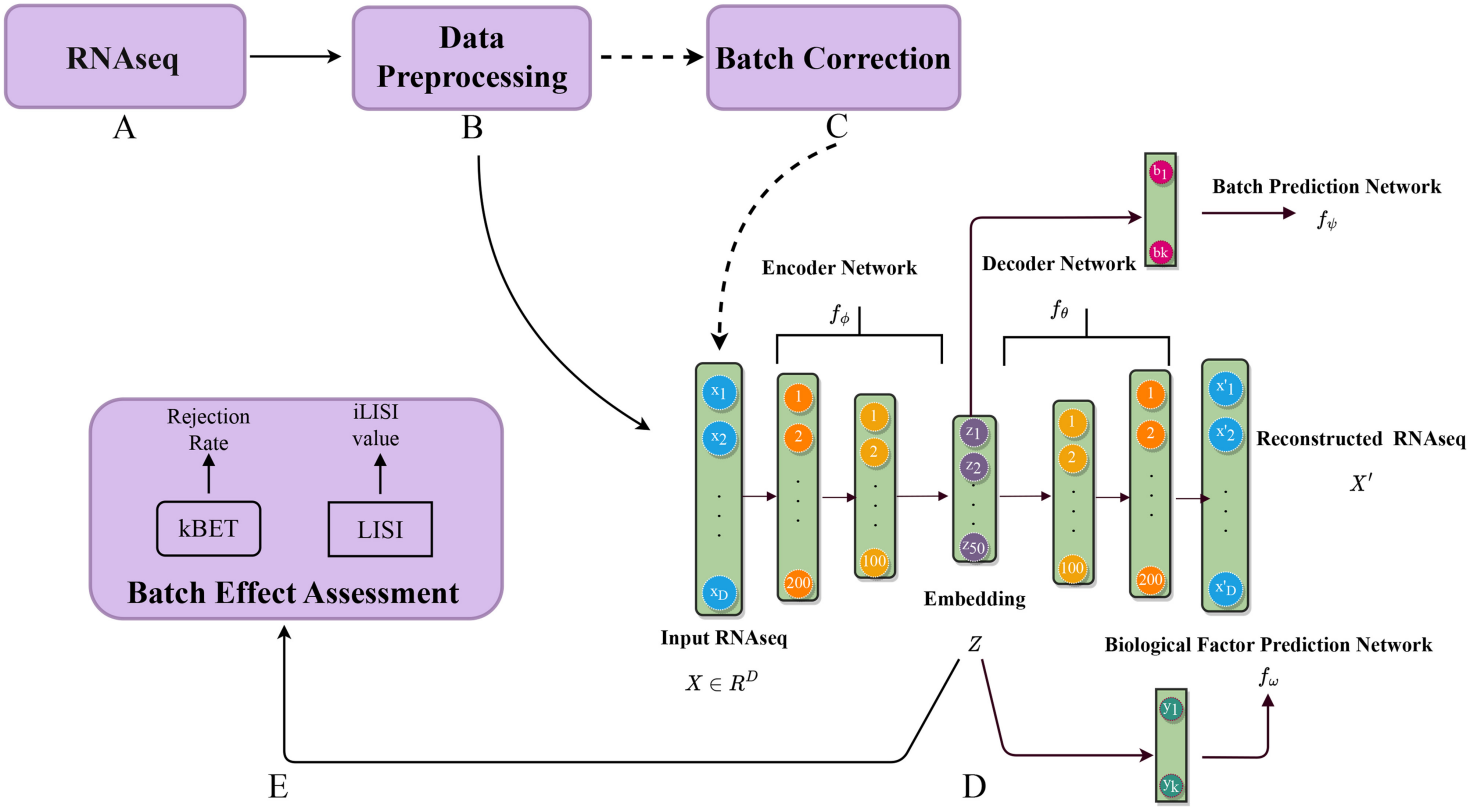
(A–E) Workflow of BEENE. BEENE takes as input RNA-seq data (before or after batch correction) and produces an embedding *Z*, which can subsequently be used by batch estimation methods, such as kBET and LISI. (D) The auto-encoder model used in BEENE. The model consists of an encoder-decoder network ($f_{\phi }-f_{\theta }$), one batch prediction network ($f_{\psi }$), and one biological factor prediction network ($f_{\omega }$). These are jointly optimized by mini batch gradient descent.

We propose BEENE, a supervised and guided learning of nonlinear embedding to estimate batch effects in scRNA-seq data. The workflow of BEENE is illustrated in Fig. 1A–E. After preprocessing steps (e.g. normalization), BEENE uses an autoencoder model to learn the nonlinear embeddings of RNA-seq expression data, which is guided by two additional modules: (i) a network to predict batch variables (e.g. cohorts, protocols) that indicate the batches from which the RNA-seq data was generated, and (ii) a network to learn biological variables (e.g. cell types, disease status). The nonlinear embedding learned by the autoencoder is used by both batch and biological variable learner modules. The autoencoder and these two learning networks are trained in tandem to guide the embedding in such a way that biological heterogeneity in the data as well as variability across batches are preserved. The batch learner module learns to predict the known and unknown probable batches across datasets and the biological learner module learns to predict the cell types in single-cell data and/or any other phenotypic information available in the samples. Thus, these two learners guide the autoencoder to learn an embedding that best explains the data in terms of their biological properties and batches, even if the batches across samples are highly nonlinear. This representation of the data is then utilized to estimate batch effects. We train the entire model, which includes the autoencoder and two additional modules, using three loss functions that are jointly optimized using the mini batch gradient descent method (see Materials and Methods for details). We investigated the efficacy of the embedding produced by BEENE in detecting batch effects with alternative measures: the *k*-nearest-neighbor (KNN) based local distribution of batches (kBET) (Büttner *et al.* 2019) and the local inverse Simpson’s index (iLISI) (Korsunsky *et al.* 2019). We applied kBET and LISI in both PCA space and BEENE embeddings, and found that kBET and LISI with BEENE provide more robust and sensitive estimation of batch effects than those using the PCA space, regardless of the linear or nonlinear nature of batch effects.

### 2.2 Nonlinear dimensionality reduction

BEENE uses a deep autoencoder to project a high dimensional RNA-seq data to a lower dimensional space by nonlinear transformations. An autoencoder is a type of artificial neural network that learns to copy its input to its output. This is achieved by learning efficient data embedding in an unsupervised manner to recreate the input. A schematic diagram of the autoencoder-based architecture of BEENE is shown in Fig. 1D. It consists of an encoder network and a decoder networks. The encoder network can be defined as $f_{\phi }:X\to Z$, which maps the input space $X\in R^{D}$ to the embedding space $Z\in R^{d}$ where d≪D. The decoder network can be defined as $f_{\theta }:Z\to X$, which maps the embedding *Z* back to the input space *X*. This intermediate embedding *Z* can subsequently be used with batch estimation methods such as kBET or LISI to detect batch effects. Here, ϕ and θ are the parameters of encoder and decoder network, respectively. Both the encoder and decoder networks in BEENE are fully connected neural network and following the architecture proposed in (Li *et al.* 2020) with two hidden layers. The encoder network has 200 neurons in the first hidden layer and 100 neurons in the second hidden layer, while the decoder network has 100 and 200 neurons in the first and second hidden layers, respectively. The dimension *d* of the intermediate embedding space was set to 50 to make this study comparable with kBET (Büttner *et al.* 2019). However, it can be changed based on the need of a particular application. We used Scaled Exponential Linear Unit (SELU) as the activation function, which was found superior for a wide range of tasks (Klambauer *et al.* 2017).

BEENE uses two additional single-layer networks $f_{\psi }$ and $f_{\omega }$ to guide the embedding *Z* learned by the encoder network $f_{\phi }$ such that the embedding preserves both biological and batch-specific variations. The batch prediction network $f_{\psi }$ uses softmax or logistic regression (depending on the number of classes) to predict the batch variables associated with the input *X* from the embedding *Z* learned by encoder $f_{\phi }$. Similarly, $f_{\omega }$ predicts the biological variables associated with input *X*. $f_{\psi }$ guides the encoder network $f_{\phi }$ to learn an embedding *Z* such that the RNA-seq data will be grouped or clustered by their respective batches. $f_{\omega }$ does the same for the biological variables. As a result, the encoder network $f_{\omega }$ tries to learn an embedding *Z* that best explains the data with respect to their batches and biological variables. Moreover, this simultaneous multitask learning (i.e. predicting both batches and biological variables from the embedding *Z*) improves generalization by *inductive transfer* (Caruana 1997).

Let the input RNA-seq dataset $X=\{x_{1},x_{2},\ldots ,x_{n}\}$ with *K* batches and *M* biological classes $\left\{(x_{1},b_{1},y_{1}\right)$, $\left(x_{2},b_{2},y_{2}\right)$, $\ldots ,(x_{n},b_{n},y_{n})\}$, where $x_{i}\in R^{D}$ is an RNA-seq, $b_{i}\in \{0,1,\ldots ,K-1\}$ is the corresponding batch variable, and $y_{i}\in \{0,1,\ldots M-1\}$ is the biological variable associated with RNA-seq $x_{i}$. Let $Z=\{z_{1},z_{2},\ldots ,z_{n}\}$ be the embeddings learned by the autoencoder network, where $z_{i}\in R^{50}$. For K≥2, the batch predictor network $f_{\psi }$ performs a softmax regression and estimates the probability $P(b_{i}=j|z_{i})$ (j∈{0,1,…K−1}) from $z_{i}=f_{\phi }(x_{i})$ using Equation 2.


(1) $$\begin{array}{cc} f_{\psi }(z_{i}) & =\left[\begin{array}{c} P(b_{i}=0|z_{i};\psi )\\ P(b_{i}=1|z_{i};\psi )\\ \vdots \\ P(b_{i}=K-1|z_{i};\psi ) \end{array}\right] \end{array}$$

(2) $$=\frac{1}{\sum_{j=0}^{K-1}\;\text{exp}(\psi^{(j)⊤}z_{i})}\left[\begin{array}{c} \;\text{exp}(\psi^{(0)⊤}z_{i})\\ \;\text{exp}(\psi^{(1)⊤}z_{i})\\ \vdots \\ \;\text{exp}(\psi^{(K-1)⊤}z_{i}) \end{array}\right]$$

Here, $\psi =[\psi^{\left(0\right)},\psi^{\left(0\right)},\ldots \psi^{\left(K-1\right)}]$ are the learnable parameters (weights) of the single layer neural network $f_{\psi }$. For K=2, i.e. when there are only two batches in the dataset, $f_{\psi }$ performs logistic regression to estimate $P(b_{i}=j|z_{i})$ for j∈{0,1} using Equation 3, where σ represents the sigmoid function.


(3) $$\begin{array}{c} P(b_{i}=1|z_{i};\psi )=f_{\psi }(z_{i})=\frac{1}{1+\text{exp}(-\psi^{⊤}z_{i})}\equiv \sigma (\psi^{⊤}z_{i})\\ P(b_{i}=0|z_{i};\psi )=1-P(b_{i}=1|z_{i};\psi )=1-f_{\psi }(z_{i}). \end{array}$$

Biological variable predictor network $f_{\omega }$ follows the same architecture to predict the biological variables from embedding *Z*. In order to prevent overfitting, we used *Dropout* (at a rate of 15%–20%) (Srivastava *et al.* 2014) and $l_{2}$-regularization. The parameters of the four networks in BEENE, {ϕ,θ,ψ, and ω}, are simultaneously optimized by minimizing the loss function J(ϕ,θ,ψ,ω) using mini batch gradient descent. $B_{k,i}$ and $Y_{k,i}$ are computed using Equation 3 when K=2 and logistic regression is performed instead of softmax regression. $\lambda_{1},\lambda_{2}$, and $\lambda_{3}$ are the weights for reconstruction loss, cross-entropy loss for batch prediction, and cross-entropy loss for biological factor prediction, respectively. These are hyper-parameters of BEENE that may affect the embedding *Z* learned by the autoencoder (see Supplementary Material). Note that $\lambda_{1}$ and $\lambda_{3}$ are related to the inherent biological properties of the RNA-seq data, while $\lambda_{2}$ is related to nonbiological factors (batch effects). If not explicitly stated otherwise, in our simulation study, we have set $\lambda_{1}=1,\lambda_{2}=2,\lambda_{3}=1$, and for datasets with no biological variables, we used $\lambda_{1}=1$, $\lambda_{2}=1$ and $\lambda_{3}=0$ (i.e. when there are no biological variables associated with the RNA-seq data $X_{i}$, $f_{\omega }$ is scraped from the model). For the mouse embryonic stem cell data, which is very high dimensional with almost 11 thousand genes per cell, we have used an extra dropout regularization (with a 70% dropout rate) at the input layer of the model to reduce the risk of overfitting. Moreover, as the mean square error increases with increasing dimensions, we have used $\lambda_{1}=0.001,\lambda_{2}=5.0$ for this particular dataset to ensure a reasonable balance between the mean square reconstruction error of the autoencoder and the cross-entropy loss for batch detection. Further discussion including a clear guideline on setting these parameters are provided in Supplementary Section 2.6 in the Supplementary Material.


(4) $$\begin{array}{c} \begin{array}{c} J(\phi ,\theta ,\psi ,\omega )\\ =\sum_{i=0}^{s}(\lambda_{1}*||x_{i}-f_{\theta }(f_{\phi }(x_{i}))|{|}_{2}^{2}-\lambda_{2}*\sum_{k=0}^{K-1}1\left\{b_{i}=k\right\}\;\text{log}\;B_{k,i}-\lambda_{3}\\ {}*\sum_{k=0}^{M-1}1\left\{y_{i}=k\right\}\;\text{log}\;Y_{k,i}+10^{-4}*\sum_{w\in \{\phi ,\theta ,\psi ,\omega \}}\left|\right|w|{|}_{2}^{2})\\ \text{Here},\\ B_{k,i}=P(b_{i}=k|f_{\phi }(x_{i});\psi )=\frac{\;\text{exp}(\psi^{(k)⊤}f_{\phi }(x_{i}))}{\sum_{j=0}^{K-1}\;\text{exp}\;(\psi^{(j)⊤}f_{\phi }(x_{i}))}\\ Y_{k,i}=P(y_{i}=k|f_{\phi }(x_{i});\omega )=\frac{\;\text{exp}(\omega^{(k)⊤}f_{\phi }(x_{i}))}{\sum_{j=0}^{M-1}\;\text{exp}\;(\omega^{(j)⊤}f_{\phi }(x_{i}))}\\ 1\left\{.\right\}\;\text{is an indicator function}.\\ s\;\text{is the batch size} \end{array} \end{array}$$

### 2.3 k-Nearest neighbor batch-effect test (kBET)

The batch effect assessment metric kBET (Büttner *et al.* 2019) compares the local batch distribution with the global distribution to measure the extent of batch effect in the dataset. After reducing dimensionality by PCA (top 50 PCs by default), it selects the *k* nearest neighbors around each point and calculates the local batch distribution. Next, it selects 10% of the points to test their local distributions with the global distribution. It performs a $\chi^{2}$ test to check whether the null hypothesis, that all the batches are well mixed, can be rejected. Low rejection rates imply well-mixing of the data while higher rejection rates, i.e. rejection rates close to 1.00 indicate the presence of a significant amount of batch effects in the data. In the default settings of kBET (https://github.com/theislab/kBET), 100 rejection rates are calculated on 100 different sub-samples of the dataset.

### 2.4 Local inverse Simpson’s index (LISI)

LISI (Korsunsky *et al.* 2019) is another local method for batch estimation. But unlike kBET, it does not use any fixed value for *k* to determine the number of neighbors. It selects neighbors based on a local distance distribution with a fixed perplexity (by default 30), and uses inverse Simpson index of diversity (Korsunsky *et al.* 2019) to determine the effective number of batches present in the locality [known as “integration LISI”(iLISI)]. An iLISI score close to 1 indicates the worst score (i.e. no-mixing of batches) and increased iLISI (close to the expected number of batches) reflects increasingly improved mixing of batches.

### 2.5 Data simulation model

Please see Supplementary Section 1 in the Supplementary Material.

### 2.6 Biological datasets

We have evaluated BEENE on a collection of biological datasets, including three scRNA-seq datasets. We used the mESCs from Klein *et al.* (2015), where the authors demonstrated the droplet-based sequencing technique. The authors of kBET used two technical replicates of this mESCs dataset that consisted of 5952 cells from 2 batches and 11 308 genes with at least 2 cells having more than 4 unique molecular identifier (UMI) reads per cell. We obtained these two technical replicates from the authors of kBET and considered these two replicates as two batches. Thus, this dataset represents a model condition with two batches where all the cells are from a single cell type (embryonic stem cell). We have used three human PBMC datasets from Korsunsky *et al.* (2019), that were assayed on the Chromium 10X platform but prepared with different protocols: 3ʹ end v1 (3pV1), 3ʹ end v2 (3pV2), and 5ʹ end (5p). These different protocols were considered as different batches and there were seven cell types considered as biological variables. We used another scRNA-seq dataset from Korsunsky *et al.* (2019) that consisted of 14 746 human pancreatic islet cells from 36 donors, assayed on 5 independent studies: inDrop (4 donors, 8569 cells), Fluidigm C1 (13 donors, 638 cells), Smart-Seq2 (10 donors, 2355 cells), CEL-seq (5 donors, 946 cells), and CEL-seq2 (4 donors, 2238 cells). Thus, we have used datasets from both human and mouse with varying conditions (e.g. number of cells, cell-types, technological platforms).

## 3 Results

### 3.1 BEENE effectively captures batch effects

**Figure 2.**
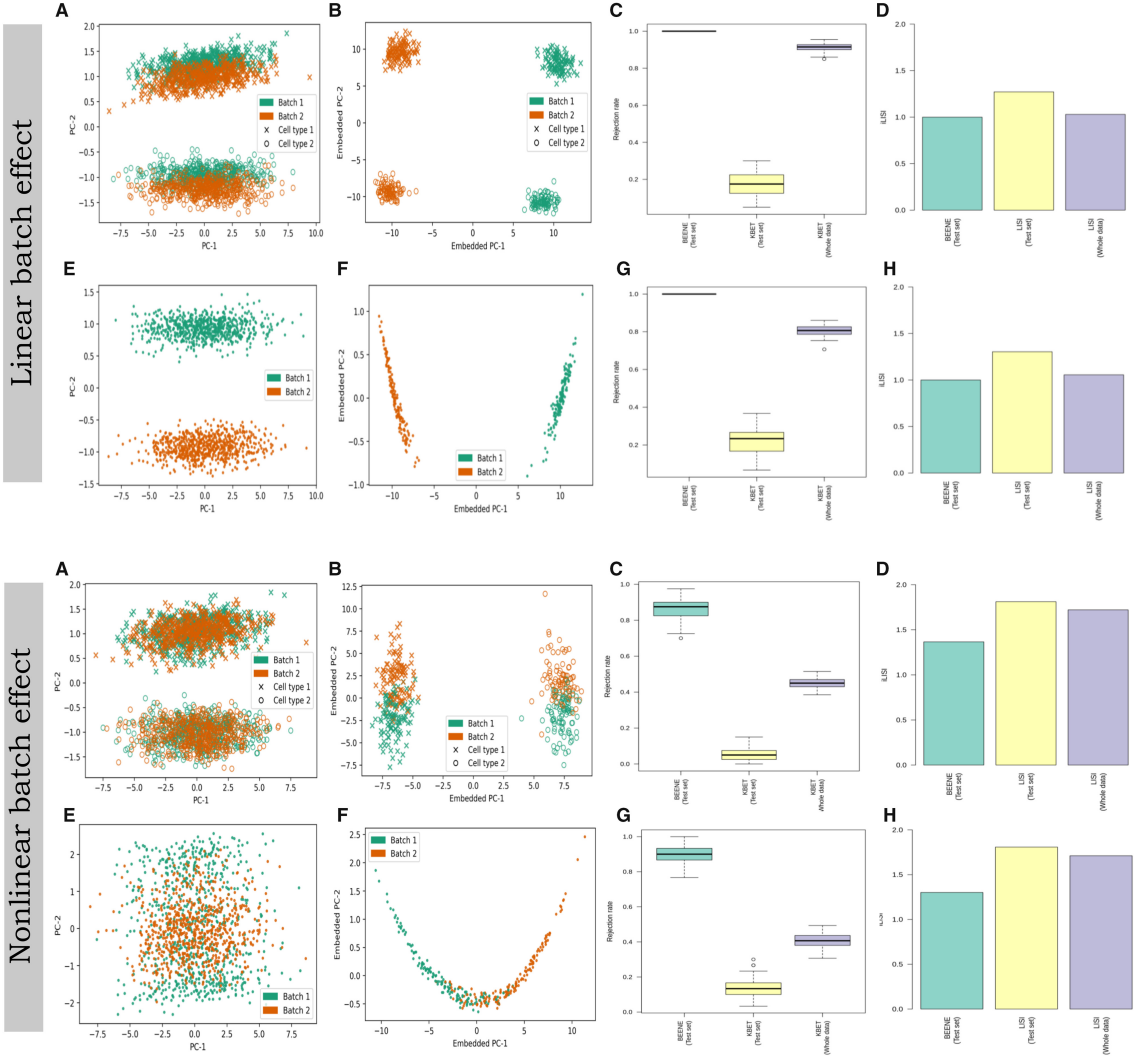
Performance of BEENE on simulated scRNA-seq data with linear and nonlinear batch effects (shown in top and bottom panels respectively). (A) scRNA-seq data from two cell types in the PCA space. (B) scRNA-seq data from two cell types in the embedding space produced by BEENE. We used the first two PCs to visualize the data embeddings. (C) Rejection rates provided by kBET in the BEENE embedding (on the testset) and in the PCA space (on both the testset and the entire dataset). In each case, we show the distribution of 100 rejection rates provided by kBET. (D) iLISI values obtained using BEENE and PCA embeddings. (E–H) Similar to A–D using one cell type.

To test the efficacy of BEENE, we used simulated scRNA-seq data generated by Splatter R package (Zappia *et al.* 2017). We have simulated expression data in 2 different settings: (i) scRNA-seq data for two different cell types where 1000 cells (with 2500 genes each) were sampled from each of these two cell types and, for each of these two cell types, two batches were generated (each with 500 cells); and (ii) scRNA-seq for a single cell-type, where 1500 cells with 2500 genes each were sampled and two batches were generated (each with 750 cells). In both settings, expression data of these two batches have been generated with additive linear noise. Next, to train (and test) the model of BEENE, we divide the expression data randomly into training, validation, and testing sets with a 60:20:20 ratio. We estimated batch effects by measuring kBET and iLISI metrics in the embedding space given by BEENE on the test sets and compared them with kBET and iLISI values in the PCA space. Figure 2A–H (top panel) summarizes the assessment and the effectiveness of BEENE compared to the PCA space. For the dataset with two cell types, the batches are not clearly separated in the PCA space (Fig. 2A; top panel). The cells from different batches are closer than the cells from different cell types indicating that the dataset is either batch free or has small batch effects if we consider the shift of cells from the two batches. In case of the data with a single cell type, the expression data shows a clear separation by their respective batches, which was captured by the second PC (Fig. 2E). In contrast, the expression data in both cases shows much clearer separation by batches and cell types in the embedding space provided by BEENE, where the first PC of the embedding space captured the batch variation and the second PC captured different cell types (Fig. 2B and F). This indicates the presence of batch effects which was not effectively captured by the PCA space. We also investigated the performance of BEENE embeddings in comparison to t-SNE and UMAP embeddings—two widely used embeddings for the visual inspection of batch effects. Similar to PCA embeddings and unlike BEENE embeddings, t-SNE failed to capture the batch- and cell-specific variations (see Section 2.1 in Supplementary Material). As a result, kBET and LISI employing the BEENE embeddings achieved higher rejection rates and lower iLISI values respectively, compared to the values obtained using the PCA embeddings (Fig. 2C, D, G, and H)—indicating that BEENE has a higher sensitivity to the presence of batch effects than the PCA space.

We conducted additional experiments to evaluate the performance of BEENE in situations where biological factors, such as cell types, were not provided (see Supplementary Fig. S3). The results indicate that BEENE was still able to accurately detect batch effects even without biological information. However, it should be noted that the embeddings generated by BEENE without biological information were unable to properly distinguish data based on cell types, as shown in Supplementary Fig. S2B. This behavior was expected, given that biological factors were not provided to the method. Nevertheless, this did not adversely impact BEENE’s ability to estimate batch effects, as demonstrated by the kBET rejection rate and the iLISI value obtained by BEENE.

### 3.2 BEENE effectively captures nonlinear batch effects

We next investigated the performance of BEENE on simulated scRNA-seq data with nonlinear batch effects. We modeled the nonlinear batch effect as an additive term to the simulated scRNA-seq data generated using Splatter (see Section 2 for details) and trained our model in a similar way as described in Section 3.1. Similar to Section 3.1, we have used two settings for simulating data, one with a single cell type and the other with two cell types. In both cases, batch effects were modeled considering data from two batches. The bottom panel in Fig. 2A–H demonstrates the effectiveness of BEENE in detecting and assessing nonlinear batch effects in comparison to the PCA space. Nonlinear batch effects are not visually separable when it is inspected in the PCA space (Fig. 2A and E; bottom panel). For data with one cell-type, one batch is more concentrated at the center without any clear separation between two batches and thus kBET gives lower rejection rates and LISI gives higher iLISI values in general, despite the presence of batch effects that was carefully introduced in our simulation study (Fig. 2C, D, G, and H; bottom panel). In contrast, the presence of batch effects is clearer in the embedding space generated by BEENE. For data with two types of cells from two batches, the BEENE embedding space effectively separates the data by both batches and cell types, where the batch variation is captured by the second principal component of the embedding space (Fig. 2B). Similarly, for the data with a single cell type, the first principal component of the embedding space captures the batch variation while the second one focuses on the biological variability of cells (Fig. 2F). In both cases, kBET in the embedding space gives higher rejection rates (Fig. 2C and G) and LISI gives lower iLISI values (Fig. 2D and H) compared to those obtained using the PCA space, indicating that BEENE has a higher sensitivity than the PCA space for detecting nonlinear batch effects.

### 3.3 BEENE does not overestimate batch effects

We have demonstrated in Sections 3.1 and 3.2 that the embedding produced by BEENE can capture batch variations more effectively than the PCA space. As a result, BEENE is more sensitive to the presence of batch effects and kBET and LISI measured on BEENE embedding resulted in higher rejection rates and lower iLISI values than those computed using the PCA space in the presence of batch effects. To investigate whether this is an indication of overestimation of batch effects, we tested our framework on two carefully controlled simulated datasets, using identical settings as Sections 3.1 and 3.2 but without introducing any batch effects. These datasets represent model conditions where data has been sequenced using two different technologies but no technical variation has been introduced into the data. In both cases, BEENE embeddings lead to rejection rates close to 0.0 and iLISI values close to 2.0, indicating no potential batch effects. kBET and LISI using the PCA space, achieved comparable yet higher rejection rates and lower iLISI values (see Supplementary Fig. S5). For data with two cell types, rejection rate drops from 0.85 to 0.0 and iLISI value increases from 1.36 to 1.95 when data is changed from batch-affected (nonlinear) to batch-free model conditions. In case of data with a single cell type, the rejection rate drops from 0.87 to 0.0 and the iLISI value increases from 1.32 to 1.95. Therefore, BEENE estimates the batch effects with necessary *specificity*. To show the robustness of batch assessment scores obtained from BEENE embeddings, we have also performed 5-fold cross-validatio (Kohavi *et al.* 1995) for nonlinear batch affected and batch free model conditions (see Supplementary Material). Furthermore, these results indicate that the batch estimations of BEENE described in Sections 3.1 and 3.2 are not overestimations, but instead represent appropriate sensitivity to batch effects. In sum, BEENE embedding outperforms PCA space in terms of sensitivity and specificity for detecting batch effects.

### 3.4 Sensitivity of BEENE to different levels of batch effects

We have assessed the performance of BEENE on simulated data with different levels of batch effects. Levels of batch effects were controlled by confining the components of the vector representing batch effects within a certain range *[*0, *b]* by choosing suitable values of *A* in Equation 2 in the Supplementary Material (see Supplementary Section 1 in Supplementary Material for details). Denoting the batch effect in the *i*th batch by a *D* dimensional vector $B^{\left(i\right)}\in R^{D}$ (*D* is the number of genes), we choose suitable values for *A* in Equation 2 in the Supplementary Material so that every component of $B^{\left(i\right)}$, $B_{j}^{\left(i\right)}\in [0,b],\forall i\in \{0,1,\ldots K-1\},\forall j\in \{0,1,\ldots ,D-1\}$. Here *[*0, *b]* is a predefined range in which higher values of *b* indicate higher levels of batch effects.

The performance of BEENE on different ranges of batch effects (*[*0, *b]*, b∈{0.1,0.2,0.3,0.4,0.5}) is shown in Supplementary Fig. S6. As expected, the rejection rates obtained using the embedding space produced by BEENE increase with increasing levels of batch effects. With a substantial level of batch effects (*[*0, 0.5*]*), the clustering based on the biological factor (cell-type) is not observed. Moreover, due to the nonlinear nature of the batch effects, the batches are not distinguishable in the PCA space but the BEENE embedding effectively separates the data by batches. Furthermore, the rejections rates of kBET using PCA space do not change appropriately with varying levels of batch effects. Even with a very high level of batch effects (*[*0, 0.5*]*), the rejection rate was around 0.4, and it did not vary significantly across a wide range of batch strengths (e.g. from *[*0, 0.2*]* to *[*0, 0.5*]*). In contrast, kBET with BEENE produced more meaningful rejection rates and it varied from 0 to 0.86 on different levels of batch effects, indicating appropriate sensitivity to varying levels of batch effects. Similar trends were observed for iLISI values, which gradually decreased from 1.90 to 1.65 with PCA space, but varied across a wider range (1.26–1.95) when BEENE embedding was used (see Table 1).

**Table 1.** Performance of BEENE on varying levels of batch effects.^a^

Amplitude range	LISI	BEENE + LISI	BEENE + kBET	kBET
[0.0, 0.5]	1.65	1.26	0.86	0.45
[0.0, 0.4]	1.72	1.36	0.85	0.45
[0.0, 0.3]	1.78	1.59	0.65	0.19
[0.0, 0.2]	1.83	1.71	0.30	0.03
[0.0, 0.1]	1.88	1.90	0.05	0.03
[0.0, 0.0]	1.90	1.95	0.00	0.04

a We show the kBET metric (rejection rate) and iLISI values obtained by kBET and LISI using default setting (i.e. PCA embedding) and BEENE embedding.

### 3.5 Sensitivity of BEENE to the proportion of genes having batch effects

We have investigated the performance of BEENE with varying proportions (10%–100%) of genes having batch effects (see Supplementary Fig. S7). The lower the proportion of affected genes, the less tightly clustered the data based on their batches. As we increase the proportion of genes having batch effects, the clustering of the data by batches becomes evident in the BEENE embedding space. However, due to the nonlinearity of the batch effects, no segregation with respect to batches was observed in the PCA space. The rejection rates of kBET with PCA embedding varies from 0.03 to 0.45 as we increase the proportion of genes having batch effects from 10% to 100%. However, kBET with BEENE embedding demonstrated improved sensitivity, as the rejection varied across a much wider range 0.05–0.85 (see Table 2). Similar trends were observed for iLISI values.

**Table 2.** Rejection rates and iLISI values obtained by kBET and LISI using default settings (i.e. PCA embeddings) and BEENE embeddings for different percentages of genes having batch effects.

Proportion (%)	LISI	BEENE + LISI	kBET	BEENE + kBET
100	1.72	1.36	0.45	0.85
80	1.77	1.50	0.40	0.79
60	1.81	1.58	0.38	0.76
40	1.87	1.79	0.28	0.73
20	1.88	1.88	0.10	0.40
10	1.89	1.94	0.03	0.05

### 3.6 BEENE performs well on single-cell RNA-seq data

We evaluated the performance of BEENE on a collection of scRNA-seq data used in previous studies (Klein *et al.* 2015, Korsunsky *et al.* 2019) for batch effect estimation and correction including (i) two technical replicates of mouse embryonic stem cell (mESC), (ii) three human PBMC datasets assayed on the same platform with three different protocols indicating three batches, and (iii) human pancreatic islet cells from five independent studies with different technologies indicating five batches (see Section 2 for more details). These datasets were selected to cover a wide range of model conditions for batch effects of different levels and complexities.

**Figure 3.**
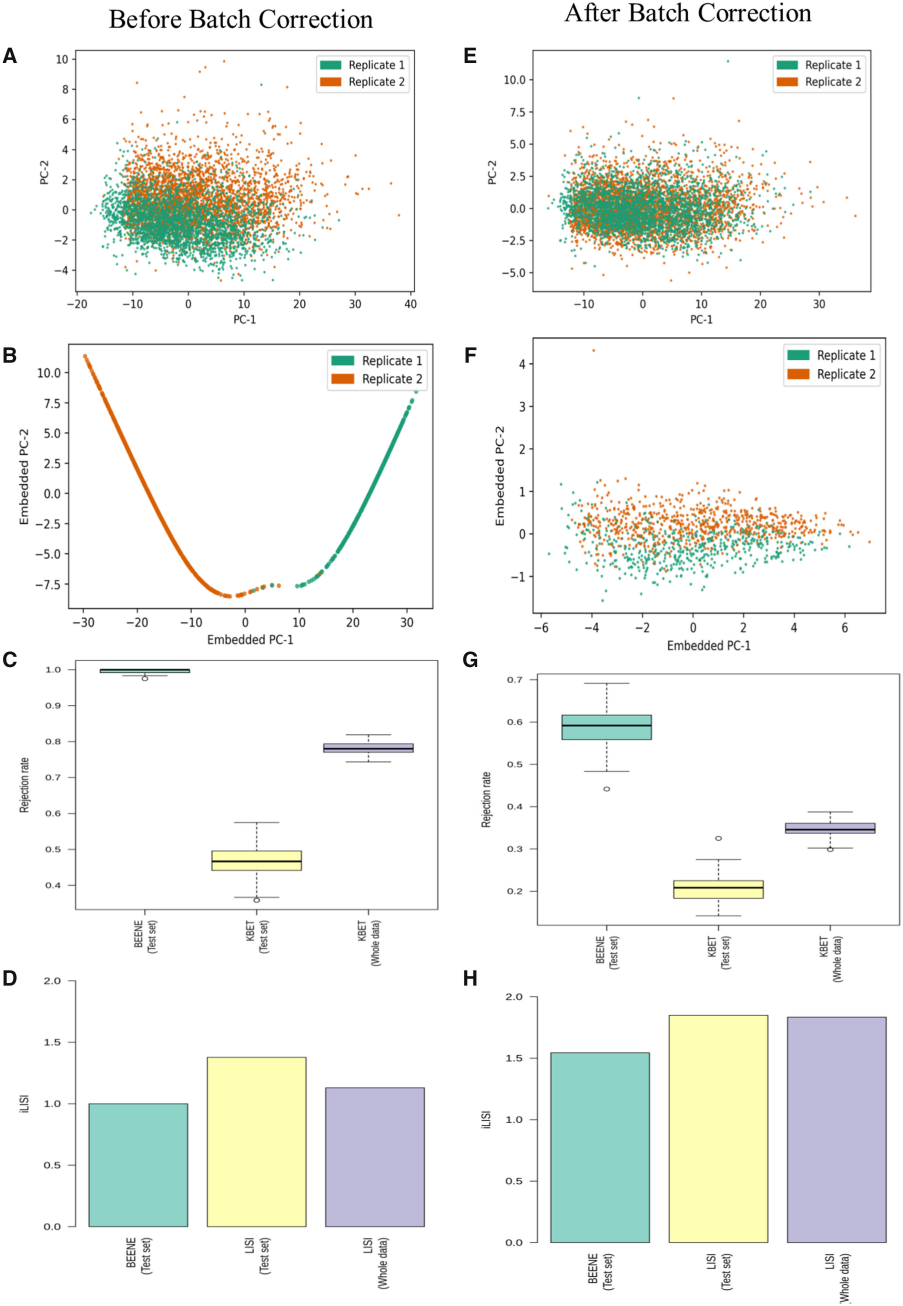
Performance of BEENE on mESC inDrop technical replicates. Results are shown for both before and after the data is batch corrected by ComBat. (A) PCA plot of the data colored by batches (technical replicates). (B) PCA plot of the embeddings learned by BEENE colored by batches. (C) Rejection rates provided by kBET using BEENE and PCA embeddings, (D) iLISI values obtained using BEENE and PCA embeddings. (E–H) Similar to A–D after batch correction by ComBat.

Figure 3 presents the comparative analysis of kBET in the PCA space and BEENE embedding on mESC data of day 0 culture generated via inDrop protocol. This represents a model condition of data from different batches with no biological variables. Before any batch correction, the batch effect is more understood in the embedding space (Fig. 3B) than in the PCA space. As a result, kBET with BEENE embedding achieved a higher rejection rate (1.00) than kBET using PCA space which obtained rejection rates of 0.80 and 0.48 on the full and test data, respectively (see Fig. 3C). Similar trends were observed for the iLISI values (Fig. 3D), suggesting that before any batch correction, batch effects are detected in both the PCA and embedding spaces, although in the embedding space it is more pronounced than the PCA space. Next, we used ComBat (Johnson *et al.* 2007), a state-of-the-art method for batch correction, to remove batch effects from the data and subsequently applied batch effect estimation methods on the corrected data. As expected, the corrected data shows reduced levels of batch effects both in the PCA space (Fig. 3E) and the embedding space (Fig. 3F). However, the embedding generated by BEENE shows a shift along the second principal component, capturing the uncorrected leftover batch effects. Consequently, BEENE achieved a higher rejection rate (0.58) than the PCA space (0.33 and 0.20 on the full test dataset respectively) (Fig. 3G) and the iLISI values for the embedding space (1.57) is closer to 1 than that for the PCA space (∼1.76). Based on our results presented in Sections 3.3 and 3.4 that BEENE embedding is sensitive to batch effects and appropriately varies with varying levels of batch effect strengths, we believe that the higher rejection rates and lower iLISI values than those using the PCA space appropriately reflect the level of the batch effects that might have persisted in the data even after the batch correction by ComBat. Thus, BEENE embedding detected batch effects with appropriate sensitivity and more clearly than the PCA space before and after batch correction.

**Figure 4.**
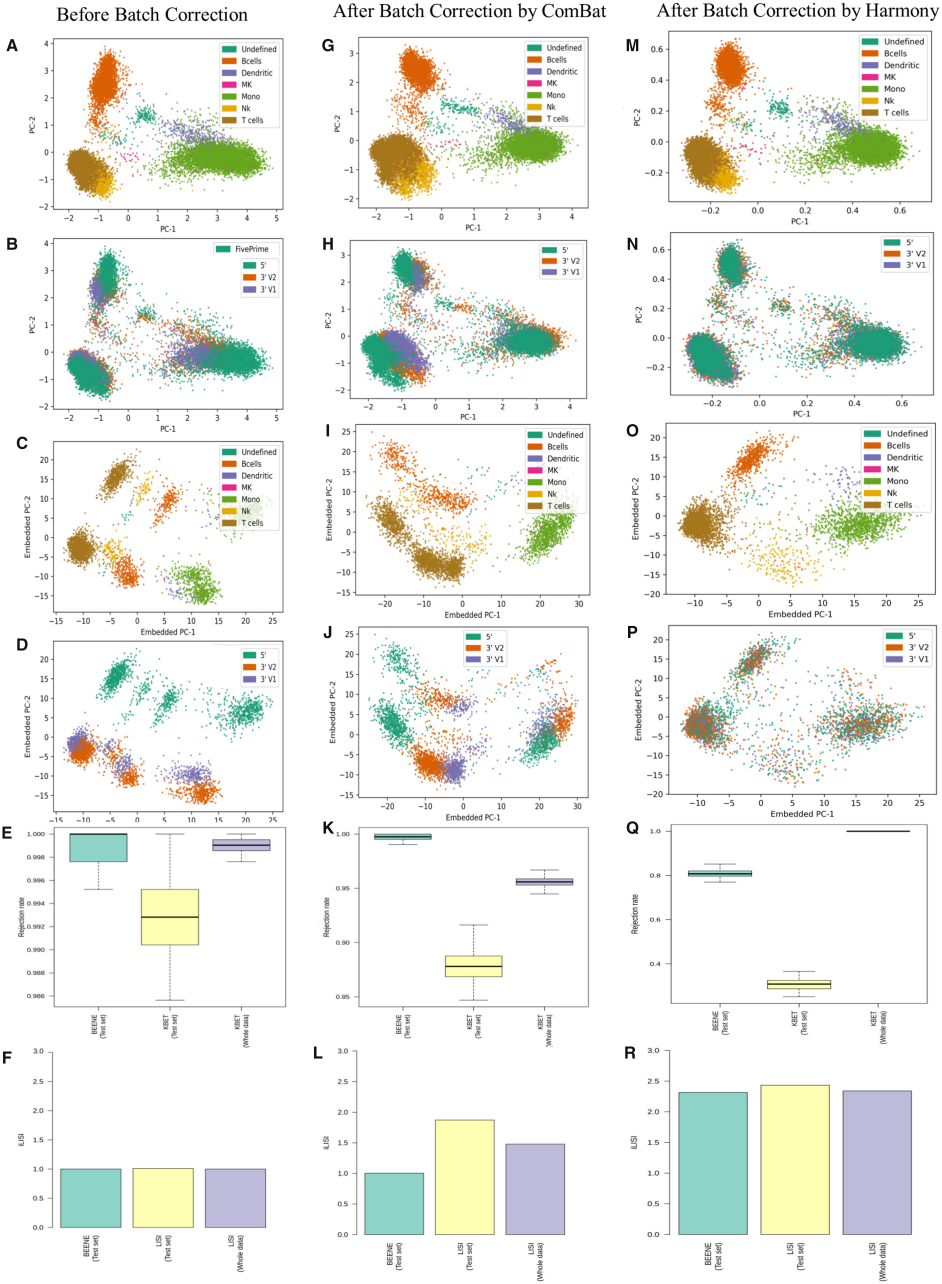
Performance of BEENE on human PBMC datasets. Results are shown before and after the data is batch corrected by ComBat and Harmony. (A) PC plot of the data colored by cell types. (B) PC plot of the data colored by batches. (C) PC plot of the embeddings learned by BEENE colored by cell types. (D) PC plot of the embeddings learned by BEENE colored by batches. (E) Rejection rates provided by kBET using BEENE and PCA embeddings, (F) iLISI values obtained using BEENE and PCA embeddings. (G–L) Similar to A–F after batch correction by ComBat. (M–R) Similar to A–F after batch correction by Harmony with the first 100 principal components of the data.

Next, we have demonstrated the performance of BEENE on three human PBMC datasets, each prepared on the Chromium 10X platform but with three different protocols. These three different protocols are considered as different batches, each with six types of cells as biological variables. In the PCA space, the cells appear to be clustered based on the biological variable, and the presence of batch-specific variations is also evident from the PCA plots (Fig. 4A and B). After applying batch correction using ComBat, the clustering remained almost similar, suggesting that ComBat may have failed to correct batch effects (Fig. 4A, B, G, and H). BEENE embedding demonstrates more clearly than the PCA embedding that the cells group by cell types and batches (Fig. 4B and C). It is also evident that the batch-specific variations remained even after batch correction (Fig. 4I and J). This is reflected in the high rejection rates before and after batch correction. Before batch correction, both BEENE and PCA embedding obtained rejection rates of close to 1.00 and after batch correction by ComBat, BEENE still gives a rejection rate of ∼1, and PCA embedding also lead to a very high rejection rate (0.96). This observation about ComBat’s failure to mix the batches well on this dataset was also demonstrated in previous studies [see Fig. 5 in Tran *et al.* (2020)]. As such, we have corrected the data by Harmony (Korsunsky *et al.* 2019) as well and investigated the performance of BEENE on Harmony- corrected human PBMC dataset. Harmony takes as input a low dimensional embedding, generally principal components, of gene expressions and returns a batch corrected embedding of the same dimension. On the other hand, BEENE takes gene expression as its input. So, in order to include Harmony, we used the first 100 principal components of PBMC data as BEENE’s input. Because the input dimension is significantly lower than that of gene expression data, we made a few changes to our model (e.g. we used only one hidden layer with 50 neurons and a 20 dimensional latent embedding for the autoencoder). Harmony-corrected data shows better mixing of batches while retaining biological variability (no mixing of cell types) both in the PC space and in the embedding space (Fig. 4M–P). This is also reflected in the rejection rate using BEENE embedding which drops to 0.80 after correction by Harmony, which was close to 1.00 for uncorrected and ComBat-corrected data. Similar trend was also noticed for iLISI values on BEENE embedding which increased from 1 to ∼2.5, indicating better mixing of the batches. This observation that Harmony performed significantly better than ComBat on this dataset is also supported by previous studies (Korsunsky *et al.* 2019, Tran *et al.* 2020). kBET and LISI using PC space, on the other hand, show contradictory trends—the iLISI values increased from 1 to 2.5 indicating mixing between batches, whereas the rejection rate of kBET remained the same even after correction by Harmony (Fig. 4Q). Thus, BEENE embedding was found to be more robust than the PCA embedding in capturing the extent of batch effects present in the data before and after batch correction. We further evaluated the performance of BEENE emabeddings on the data corrected by Combat-Seq and MNN correct. The results, presented in  Section 2.3 in the Supplementary Material, show that BEENE performs well on the data corrected by these methods as well. Additionally, we ran Combat-seq with and without biological information to assess the performance of BEENE on different model conditions. These results suggest that the rejection rate drops and the iLISI value increases more prominently on Harmony-corrected data than the data corrected by ComBat, ComBat-Seq, and MNN—indicating that Harmony was more effective in removing batch effects than other methods on this dataset. Furthermore, ComBat-Seq, when run without biological information, appears to be more effective in removing batch effects than when it is run with biological information. We hypothesize that the extra constraint of retaining the biological factors negatively affected the performance of ComBat-Seq.

Finally, we have tested the performance of BEENE on scRNA-seq data of human pancreatic islet cells from five independent studies, generated by five different technological platforms (Cel-seq, Cel-seq2, SMART-Seq2, Fluidigm C1, and inDrop) that represent five batches. Each of these 5 datasets have 10 different cell types as biological variables. The results on this dataset are presented in  Section 2.6 in Supplementary Material.

## 4 Discussion

Batch effects are inherently complex and difficult to estimate. While linear adjustments of batch effects may be adequate for many applications, it is important to consider various downstream analyses that involve more sophisticated methods such as machine learning models. These downstream analyses can be affected by the nonlinear remnant of batch effects. Therefore, even if linear adjustments appear to be sufficient, it is crucial for users to carefully evaluate the necessity of nonlinear adjustments depending on the specific application of the corrected data. Most of the existing approaches for batch effect detection and estimation rely on a lower-dimensional embedding of the data generated by PCA. In this study, we have proposed BEENE, which utilizes a deep autoencoder network to learn a latent embedding of the data using nonlinear transformation. Furthermore, we used two separate networks to learn batch and biological variations. We train these three networks in tandem with appropriate loss functions so that the embedding learned by the autoencoder is capable of explaining both batch and biological variations. BEENE is the first known study to consider and model the nonlinearity of batch effects in batch effect detection and estimation. Experimental results on various carefully controlled simulated datasets with both linear and nonlinear batch effects of different levels show that BEENE embeddings are more robust and sensitive than PCA embeddings. BEENE makes methods like kBET and LISI perform better in the face of complex batch effects. See Section 3 in Supplementary Material for additional discussion.

## Supplementary Material

btad479_Supplementary_Data

## Data Availability

The data underlying this article are available at https://github.com/ashiq24/BEENE.
