# Supplementary material for: BEENE: deep learning-based nonlinear embedding improves batch effect estimation
Source: Bioinformatics. 2023 Aug 10;39(8):btad479. doi: 10.1093/bioinformatics/btad479 (PMC10448987; doi:10.1093/bioinformatics/btad479)
Supplement: btad479_Supplementary_Data [file btad479_supplementary_data.pdf]

Supplementary Material for BEENE: Deep Learning based Nonlinear Embedding Improves Batch Effect Estimation

Md Ashiqur Rahman[1,2], Abdullah Aman Tutul[1,3], Mahfuza Sharmin[4,*], and Md Shamsuzzoha Bayzid[1,*]

[1]Department of Computer Science and Engineering
Bangladesh University of Engineering and Technology
Dhaka-1205, Bangladesh
[2]Department of Computer Science
Purdue University, Indiana, USA
[3]Department of Computer Science and Engineering
Texas A&M University, College Station, Texas, USA
[4]Department of Genetics
Stanford University, California, USA
[*]Corresponding author: shams_bayzid@cse.buet.ac.bd,
mhfz.sharmin@gmail.com

These supplementary materials present the details of data simulation model, additional results, supplementary figures and tables, and additional discussion.

1 Data simulation model

The batch effect in log-transformed data can be represented as additive terms shown in Equation 1 [1].

$$X = X^* + B + \epsilon \tag{1}$$

Here, $X, X^* \in^D$ are respectively the observed and true RNA-seq data, $B \in^D$ is the batch effect, and $\epsilon \in R^D$ is the random noise term which does not depend on any specific technical or environmental factors. Denoting various non-biological factors (e.g., ozone level, choice of reagents) that contribute to batch effects by $\beta \in^l$, the batch effect B can be expressed as a function of β, i.e., $B = f(\beta)$. In reality, this function can be highly non-linear.

We used Splatter [2], which is a widely used package to simulate single-cell RAN-seq data with batch effects [3, 4]. It covers a wide range of model conditions and simulates

batch effects with multiplicative factors that are applied to all genes in the cells from a batch. RNA-seq and batch effects are often modeled by Gaussian distribution [5, 6]. Gaussian mixer models are used to simulate RNA-seq of different cells, and batch effects are simulated by generating a Gaussian random vector for each batch and adding it to the RNA-seq of the corresponding batch. In these models, the function f that maps between B and β is linear and, therefore, could be inadequate to model complex non-linear associations between B and β. To take non-linearly into account, we propose a more general model for batch effects. We start with sampling β from multi-variate Gaussian distribution and then use a nonlinear function f to map β to batch effects B as shown in Equation 2.

$$
\begin{aligned}
&\beta^{(i)} \sim \mathcal{N}\left(\mu^{(i)}, \Sigma\right), \text{where } \mu^{(i)} \in^{l}, \Sigma \in^{l\times l}, i \in \{0, 1, \dots K-1\} \\
&I^{(i)} = W * \begin{bmatrix} \beta_0^{(i)P_0} \\ \beta_1^{(i)P_1} \\ \vdots \\ \beta_{l-1}^{(i)P_{l-1}} \end{bmatrix}, \text{where } W \in^{D\times l}, P_{0\dots l-1} \in \\
&B^{(i)} = \frac{1}{A} * \hat{g}(I^{(i)}) = \frac{1}{A} * \left[g(I_0^{(i)}), g(I_1^{(i)}) \dots g(I_{D-1}^{(i)}) \right], \\
&\text{where } A \text{ is a constant.}
\end{aligned} \quad (2)
$$

Here $\mu^{(i)}$ is the mean of the parameters of the i-th batch ($i \in \{0, 1, \dots, K-1\}$) and Σ is the co-variance matrix capturing the correlations among the parameters of the batch effect. $B^{(i)}$ is the batch effect in the i-th batch which is added to X^* to produce the observed RAN-seq X. Each element of $I^{(i)}$ is a higher order polynomial of $\beta^{(i)}_{0\dots l-1}$ of degree $\max(\{P_0, P_1, \dots, P_{l-1}\})$. P_is were sampled uniformly within the range [0,4], and the *sigmoid* function was used as g. The constant A acts as a scaling factor and controls the magnitude/level of the components of batch effects. μ, Σ, and W were sampled from the following distributions.

$$
\begin{aligned}
&\mu_i \sim U_l(-1, 1)^l \quad \forall i \in \{0, 1, \dots K-1\}, l = 100 \\
&W_{n,m} \sim U(-1, 1) \quad \forall n \in \{0, 1, \dots D-1\}, \forall m \in \{0, 1, \dots l-1\} \\
&\Sigma \sim S^l_{++}, \text{i.e., randomly sampled from the set of all} \\
&l \times l \text{ posfitive definite matrices, } S^l_{++}
\end{aligned}
$$

In case of linear batch effects, we set $P_0 = P_1 = \dots = P_{l-1} = 1$, the identity function was used for g, and $\mu_i \sim U_l(a_i, b_i)^l \quad \forall i \in \{0, 1, \dots K-1\}$, where $(a_k, b_k) \cap (a_j, b_j) = \emptyset$, $\forall j, k \in \{0, 1, \dots K-1\}$. We used Splatter [2] with its default parameter settings except the number of cells, the number of genes, and the number of cell types which were varied depending on various model conditions of our simulation study. Different cell types were simulated with an uniform probability i.e., each cell type has an equal number of cells. The batches are also distributed uniformly, i.e., each batch has an equal number of cells, and if different cell types are present, each batch has an equal number of cells from each cell type.

2 Additional Results

2.1 BEENE aids visual inspection of batch effects

BEENE can significantly aid visualization of high-dimensional data by projecting the gene expression data to the embedding space which is tailored to capture batch related variances. We have already demonstrated the superiority of BEENE embeddings over PCA embeddings in visual inspection of batch effects. We also investigated the performance of BEENE in comparison to another widely used embedding, t-SNE [7]. t-SNE embeddings, which is trained in a completely unsupervised manner and without explicitly considering batch-related information, may fail to capture highly non-linear batch effects (see Fig. S1). The first two t-SNE embeddings clearly failed to capture the batch effects (Fig. S1 A,C) in scRNA-seq data, whereas the first two PCs of BEENE embeddings can effectively capture the non linear batch effects (Fig. S1 B,D). Additionally, we compared BEENE with UMAP. The results are shown in Fig. S2.

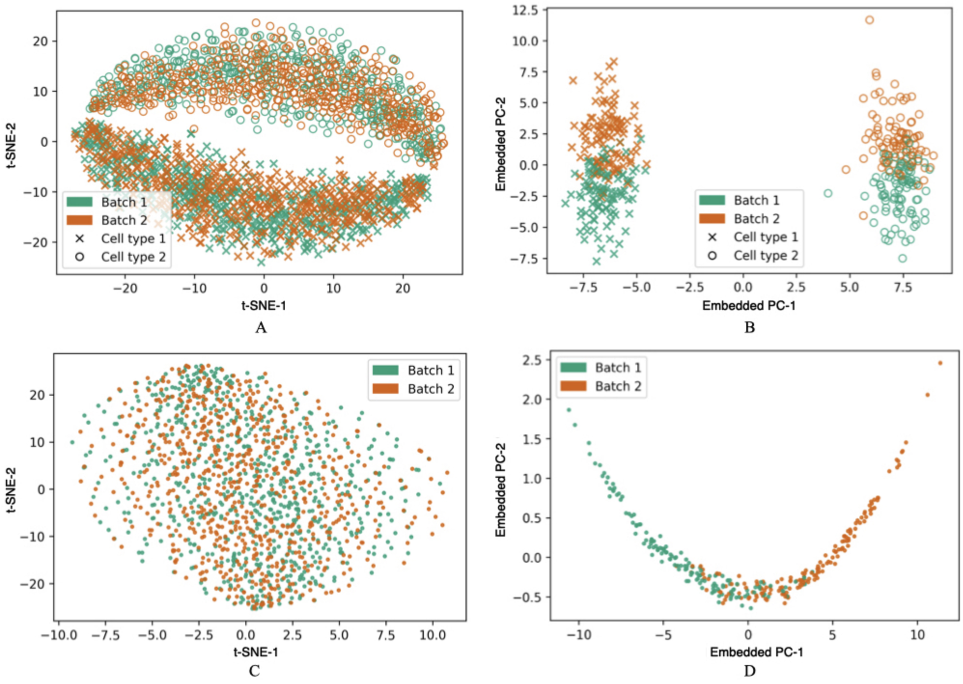

Figure S1: Visualization of non-linear batch effects on simulated data using BEENE and t-SNE embeddings. (A-B) scRNA-seq data from two cell types in t-SNE and BEENE embedding space respectively. (C-D) scRNA-seq data from single cell type in t-SNE and BEENE embedding space respectively.

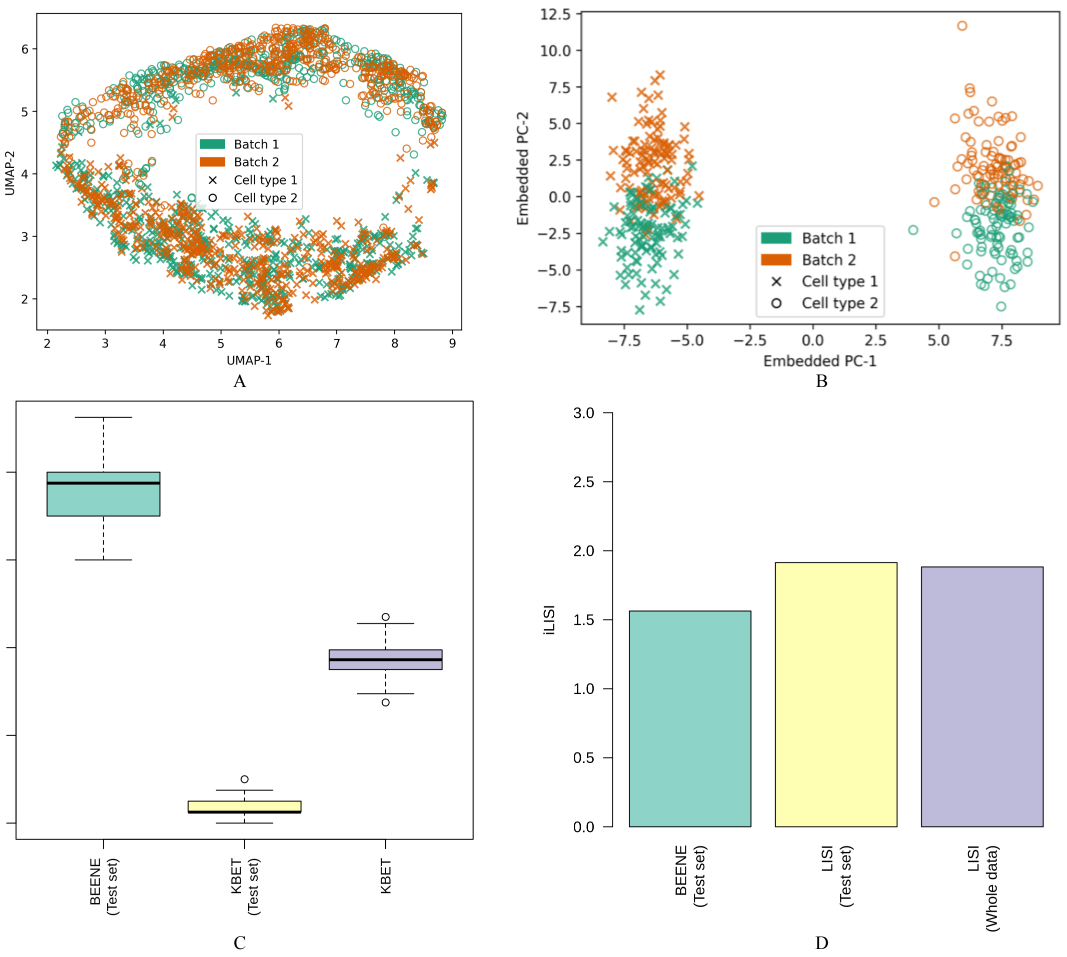

Figure S2: Performance of non-linear UMAP embeddings compared to the BEENE embeddings. (A) scRNA-seq data from two cell types in UMAP embeddings colored by batches. (B) scRNA-seq data from two cell types in BEENE embeddings (colored by batches). (C) iLISI values obtained using BEENE and UMAP embeddings. (D) Rejection rates provided by kBET in the BEENE embedding (on the testset) and in the UMAP space (on both the testset and the entire dataset). In each case, we show the distribution of 100 rejection rates provided by kBET.

2.2 Performance of BEENE with no biological information

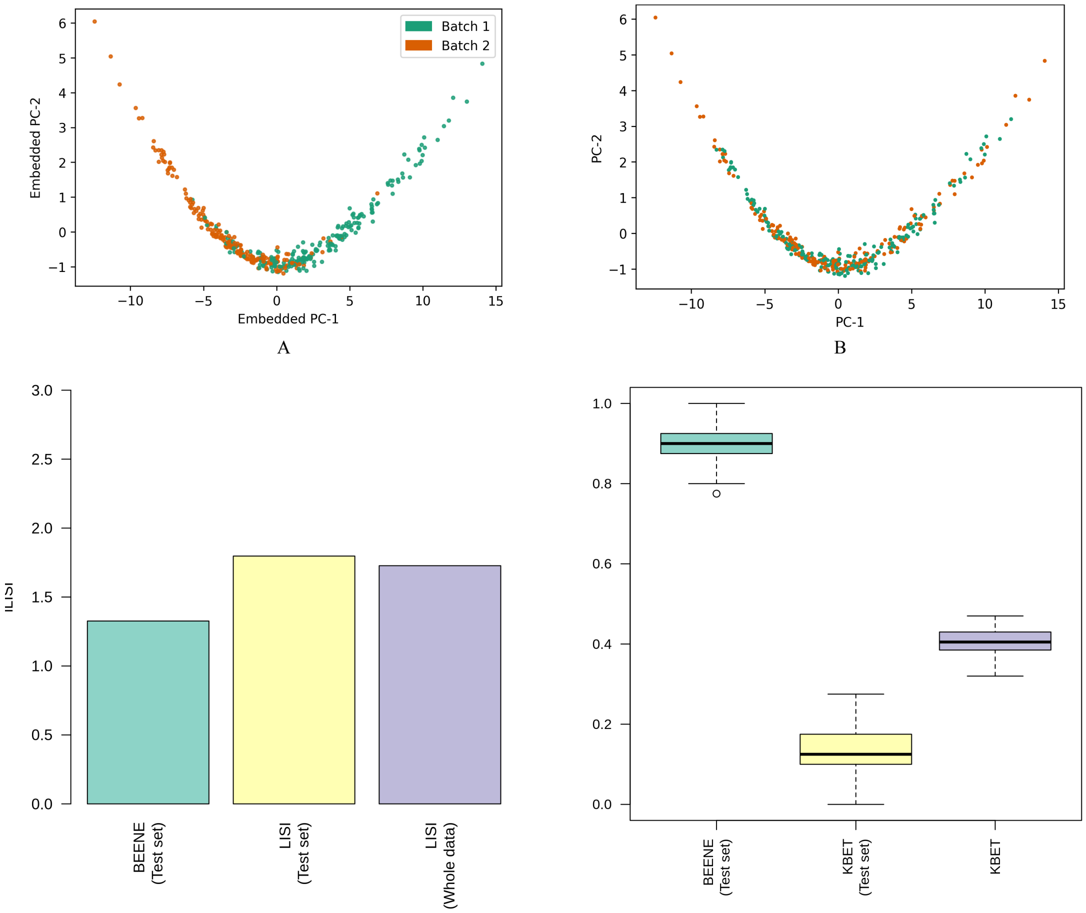

Figure S3: Performance of BEENE on simulated scRNA-seq data with nonlinear batch effects with two cell types. Here, BEENE is not provided with the biological information (i.e., cell types). (A) scRNA-seq data from two cell types in the embedding space produced by BEENE. We used the first two PCs to visualize the data colored by batches. (B) scRNA-seq data from two cell types in the embedding space produced by BEENE colored by cell types (C) Rejection rates provided by kBET when run using the BEENE embedding (on the testset) and in the PCA space (on both the testset and the entire dataset). In each case, we show the distribution of 100 rejection rates provided by kBET. (D) iLISI values obtained using BEENE and PCA embeddings.

2.3 Performance of different batch correction methods with BEENE embeddings.

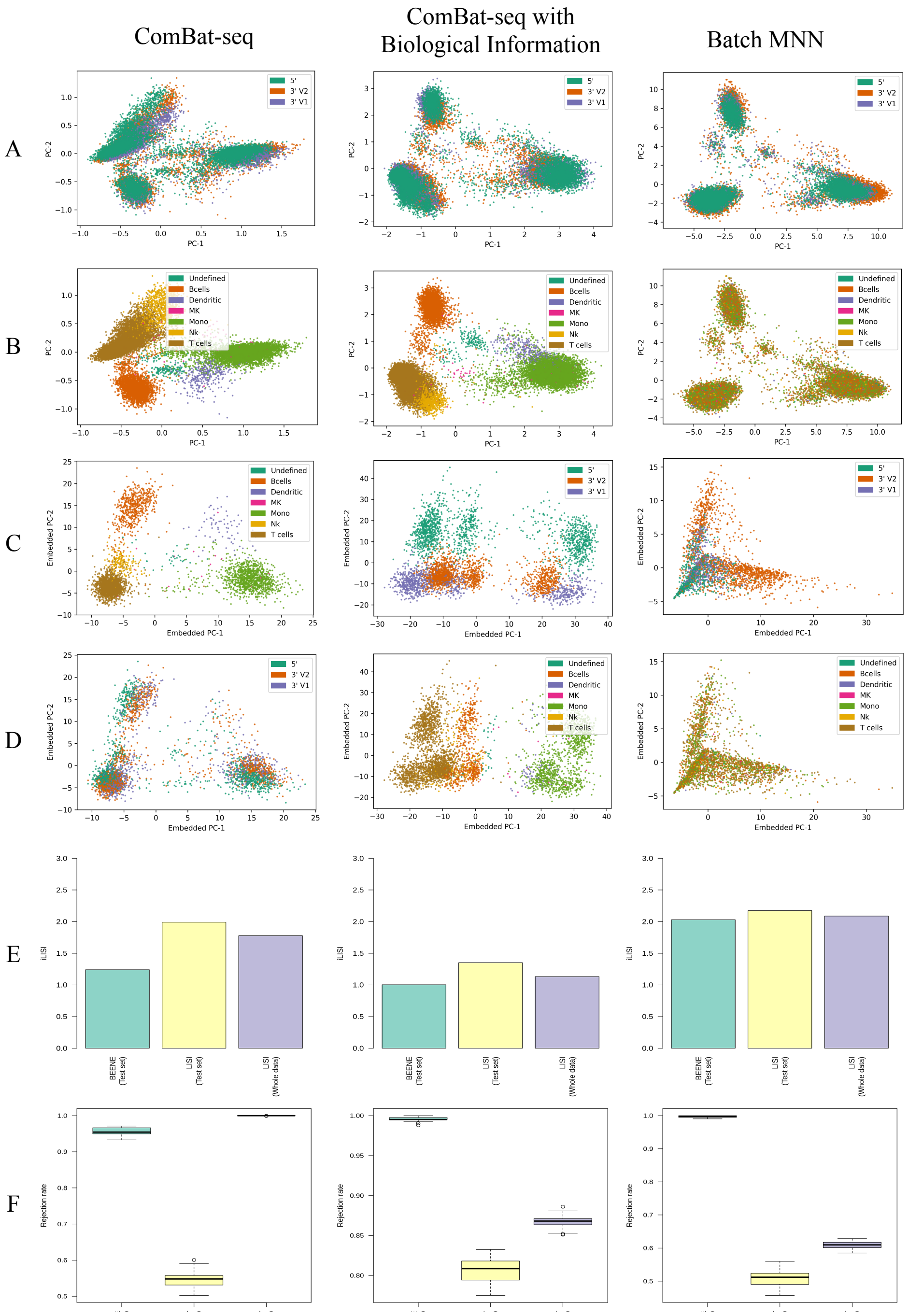

Figure S4: Performance of BEENE on human PBMC datasets. Results are shown before and after the data is batch corrected by ComBat-seq (with and without Biological information) and Batch-MNN. (A) PC plot of the data colored by batches. (B) PC plot of the data colored by cell types. (C) PC plot of the embeddings learned by BEENE colored by Batches (D) PC plot of the embeddings learned by BEENE colored by cell types (E) iLISI values obtained using BEENE and PCA embeddings. (F) Rejection rates provided by kBET using BEENE and PCA embeddings.

2.4 Additional figures

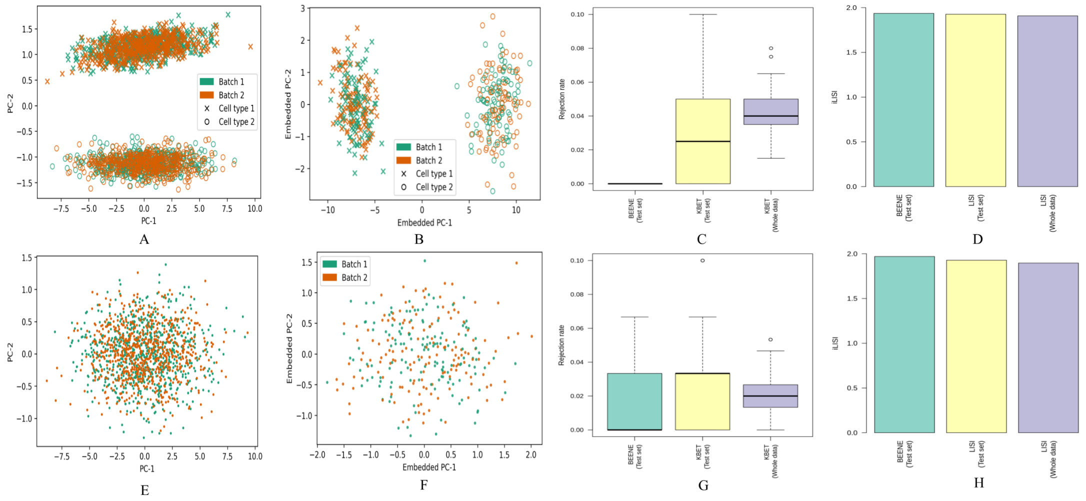

Figure S5: Performance of BEENE on simulated scRNA-seq data with no batch effects. (A) scRNA-seq data from two cell types in the PCA space, (B) scRNA-seq data from two cell types in the embedding space produced by BEENE, (C) Rejection rates provided by kBET using the BEENE embedding (on the testset) and the PCA space (on both the testset and the entire dataset), (D) iLISI values obtained using BEENE and PCA embeddings, (E-H) Similar to A-D using data with one cell type.

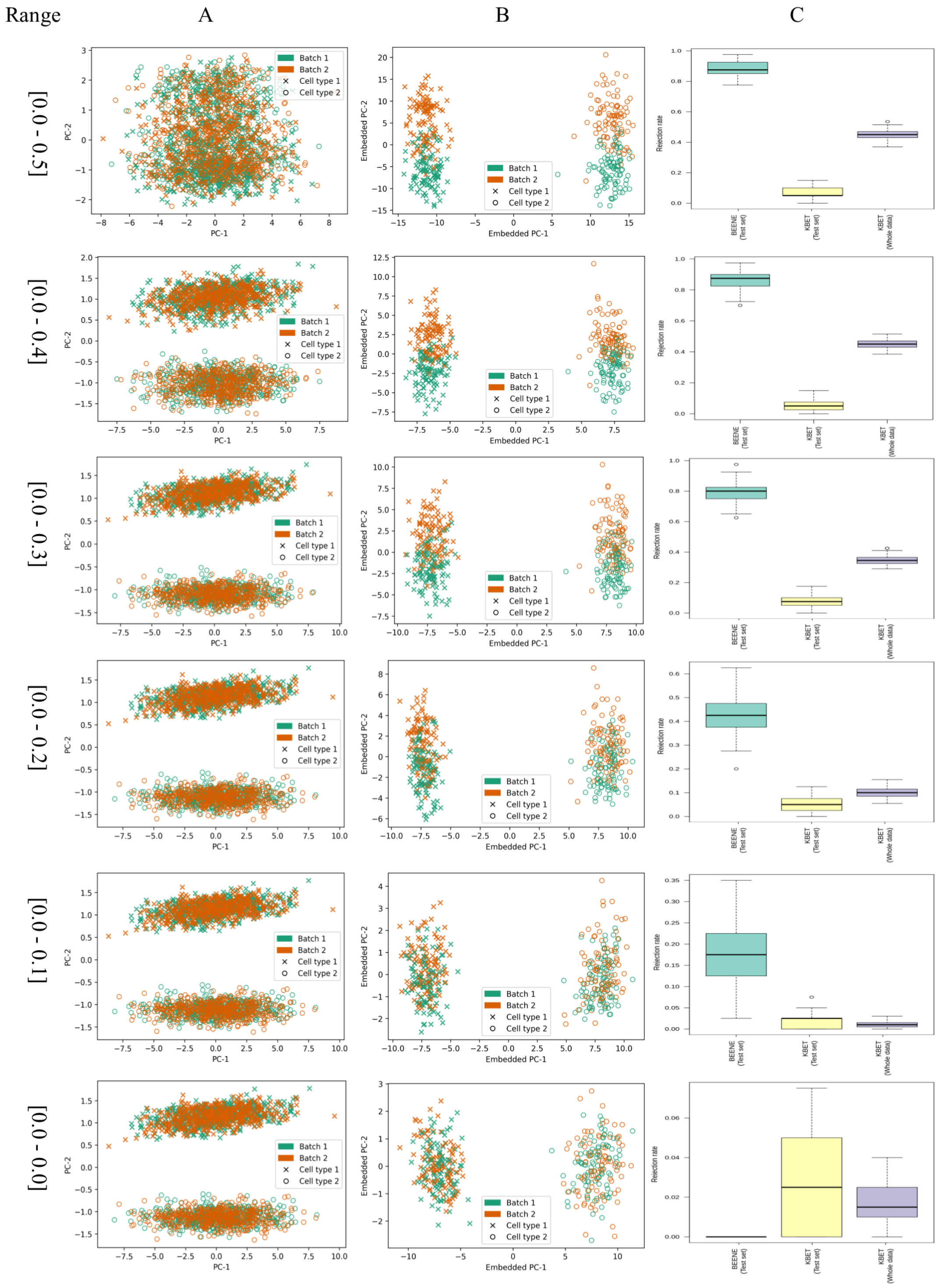

Figure S6: Performance of BEENE on simulated scRNA-seq data with varying levels of batch effects. For a particular level of batch effects, we show (A) PC plot of the data after adding batch effects, (B) PC plot of the embedding learned by BEENE, and (C) Rejection rates provided by kBET using the BEENE embedding (on the testset) and the PCA embedding (on both the testset and the entire dataset).

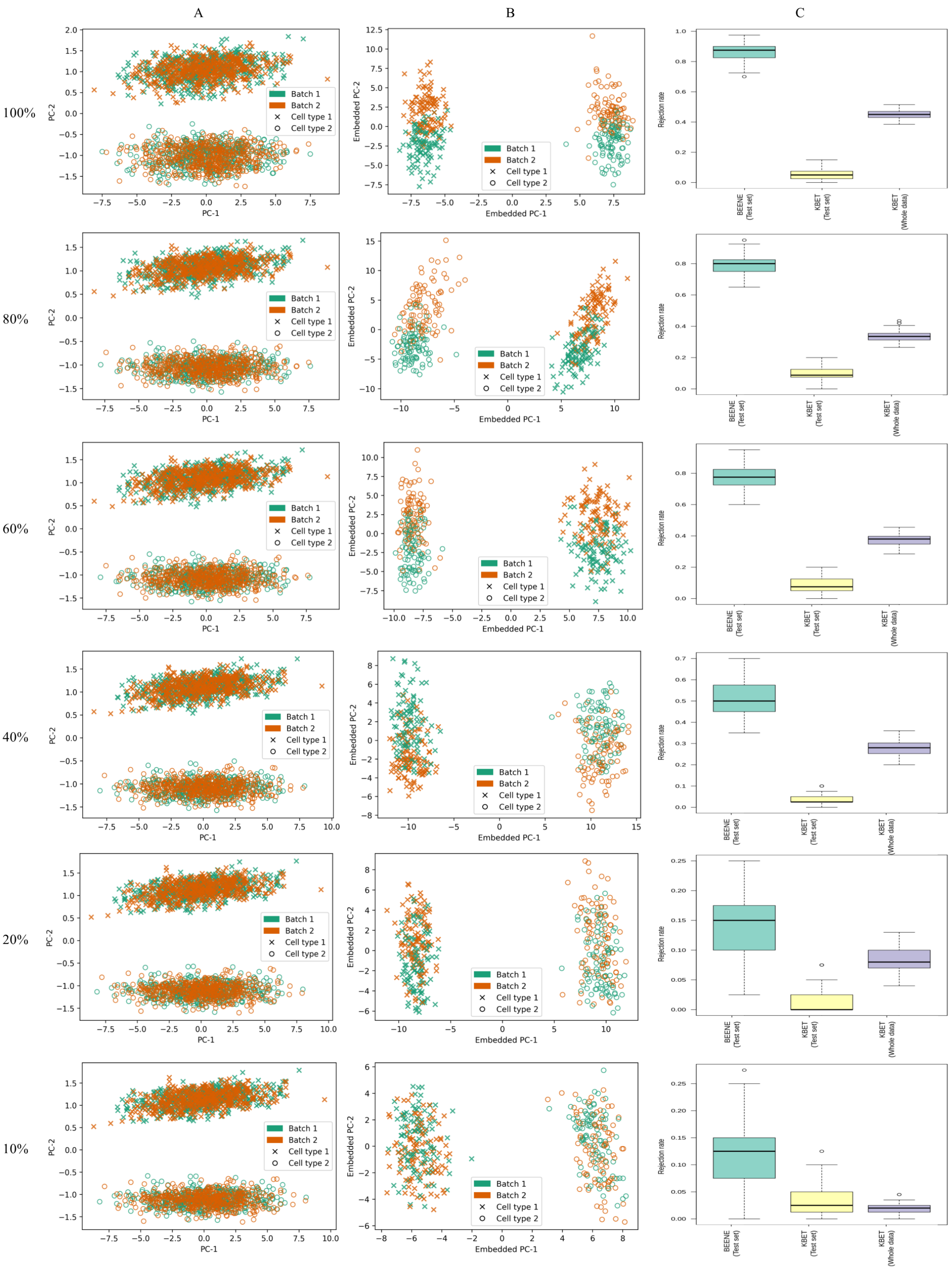

Figure S7: Performance of BEENE on varying proportion of genes with batch effects. For a particular percentage of genes with batch effects, we show (A) PC plot of the data after adding batch effects, (B) PC plot of the embedding learned by BEENE, and (C) Rejection rates provided by kBET using the BEENE embedding (on the testset) and the PCA embedding (on both the testset and the entire dataset).

2.5 Results on scRNA-seq data of human pancreatic islet cells

In the PCA space, the data becomes less tightly clustered by batches and better clustered by biological variables after conducting batch correction by ComBat than they were before any correction (Fig. S8 A-B,G-H). Similar trends were observed in the cell clustering in the BEENE embedding space. However, in the BEENE embedding space, the separation among the clusters of batches and biological variables are more pronounced than they are in the PCA space. This indicates the impact of batch correction as the biological variability becomes more dominant than the batch variability Fig. S8C-D,I-J). However, clustering by batches is also apparent after batch correction (Fig. S8J), indicating the presence of batch effects even after the batch correction. These observations are also reflected in batch assessment measurements obtained by kBET and LISI. Rejection rates of kBET with the BEENE and PCA spaces (1.00 and 0.96, respectively) remained unchanged before and after batch corrections (Fig. S8E,K), indicating that ComBat may not have sufficiently corrected the batch effects. Similar observations can be made for iLISI values (Fig. S8F,L). But the PCA plots with the first two PCs clearly failed to capture these uncorrected batch effects as the separation of the data by batches are not clear after batch correction (Fig. S8B,H,D,J). However, the BEENE embedding space is better than the PCA space in detecting batch effects as the clustering by batches is more prominent in the BEENE embedding space. Note that the iLISI value obtained using the PCA embedding increased from 1 to 1.26 on the test set but remained unchanged on the entire data set. However, the iLISI values using BEENE embeddings before and after batch correction remained the same on the test set, and thus the embeddings learned by BEENE can better portray the actual condition of batch effects in the data and can provide more accurate results on the test set which is only 20% of the whole data. It indicates that BEENE is *sample efficient* in detecting batch effects.

2.6 Impact of hyper parameters

BEENE uses three hyper-parameters λ_1, λ_2, and λ_3 (see Equation 3 in the main text), representing the weights for reconstruction loss, cross entropy loss for batch prediction, and cross entropy loss for biological factor prediction, respectively. The relative values of these hyper-parameters have an impact on the embedding obtained by BEENE as they scale the gradients of the losses. Moreover, as the model is optimized by mini batch gradient descent, the relative values of these hyper parameters determine in which direction the parameters will be moved in various iterations. We have demonstrated the impact of these hyper parameters in Fig. S9 using simulated data with two cell types from two batches.

With an increase in λ_2 relative to λ_1 and λ_3, the data becomes tightly clustered by their batches, while the clustering by the cell types disappears. For example, for $\lambda_1 = \lambda_3 = 0.01$ and $\lambda_2 = 1.00$ in Fig. S9, different batches were clearly captured but there was no separation based on the biological variability (i.e., cell types). When λ_2 is substantially higher than λ_1 and λ_3, the batch variation is captured by the first principle component of the embedding space i.e., batch variance is the dominant part of the total data variation (see the model conditions with $\lambda_1 = \lambda_3 = 0.05$, $\lambda_2 = 1.00$ and $\lambda_1 = \lambda_3 = 0.01$, $\lambda_2 = 1.00$). On the other hand, if λ_2 is substantially lower than λ_1 and λ_3, the embedding space fails to separate the data by batches but clearly captures the variability in cell types (see the

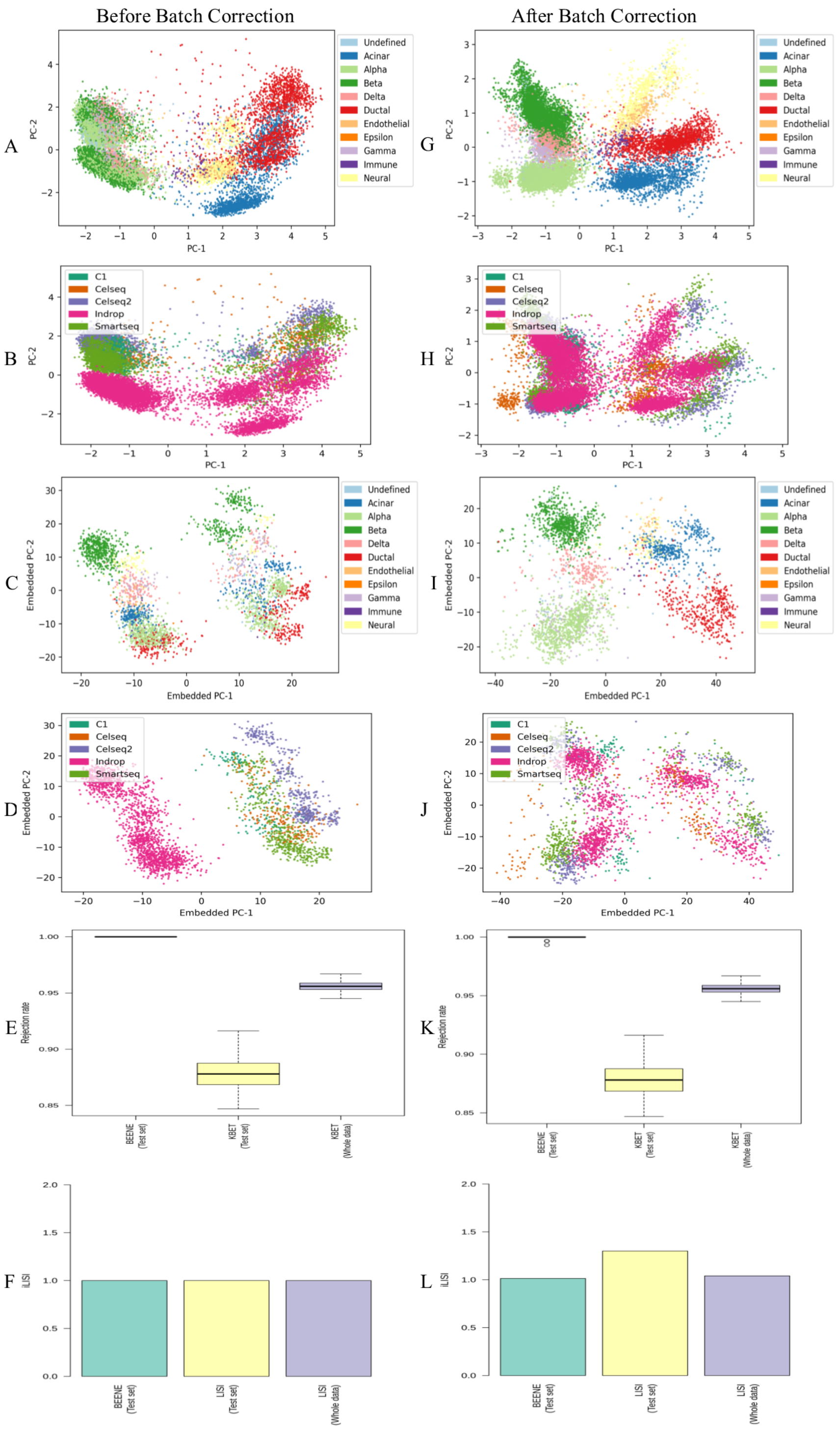

Figure S8: Performance of BEENE on pancreatic islet cells. Results are shown before and after batch correction by ComBat. (A) PC plot of the data colored by cell types. (B) PC plot of the data colored by batches. (C) PC plot of the embeddings learned by BEENE colored by cell types (D) PC plot of the embeddings learned by BEENE colored by batches (E) Rejection rates provided by kBET using BEENE and PCA embeddings, (F) iLISI values obtained using BEENE and PCA embeddings, (G-L) Similar to A-F after batch correction by ComBat.

Table S1: Recommended values for the hyper-parameters λ_1, λ_2, and λ_3 under different dataset conditions.

Biological Feature	λ_1	λ_2	λ_3	Dimensionality reduction applied?
Present	1.000	2.0	1.0	Yes (i.e., the number features is in the range of hundreds)
	0.001	5.0	5.0	No (i.e., the number features is in the range of thousands)
Absent	1.000	1.0	-	Yes
	0.001	5.0	-	No

model condition with $\lambda_1 = \lambda_3 = 1.00$ and $\lambda_2 = 0.05$). The embedding space can separate the data by both batches and cell types when λ_2 is slightly (or moderately) higher than λ_1 and λ_3 and the variations in cell types is captured by the first principle component (see the model condition with $\lambda_1 = \lambda_3 = 1.00$ and $\lambda_2 = 2.00$). This is because the inherent biological variability is expected to be more pronounced than batch effects. Therefore, setting λ_2 higher than λ_3 helps the model capture the clusters by batches, but λ_2 should not be substantially higher than λ_3 to preserve the biological variability. We provide a guideline on setting these hyper-parameters for an arbitrary dataset in Table S1. The intuition is to give equal importance on both biological variability and batch variability, and therefore the parameter values were chosen as such that each kind gets 50% weights. To be specific, choosing $\lambda_1 = 1, \lambda_2 = 2, \lambda_3 = 1$ for datasets with biological variables and choosing $\lambda_1 = 1$, $\lambda_2 = 1$, $\lambda_3 = 0$ for datasets with no biological variables effectively puts 50% weights on biological and non-biological variability. The exception to this is when, due to no dimensionality reduction (e.g., PCA), the number of features are extremely large (as in the mESC dataset). For a high-dimensional data with number of features in the range of thousands, the reconstruction loss is chosen very low (0.001) compared to rest of the hyper parameters to account for the curse of dimensionality and other related issues thereof.

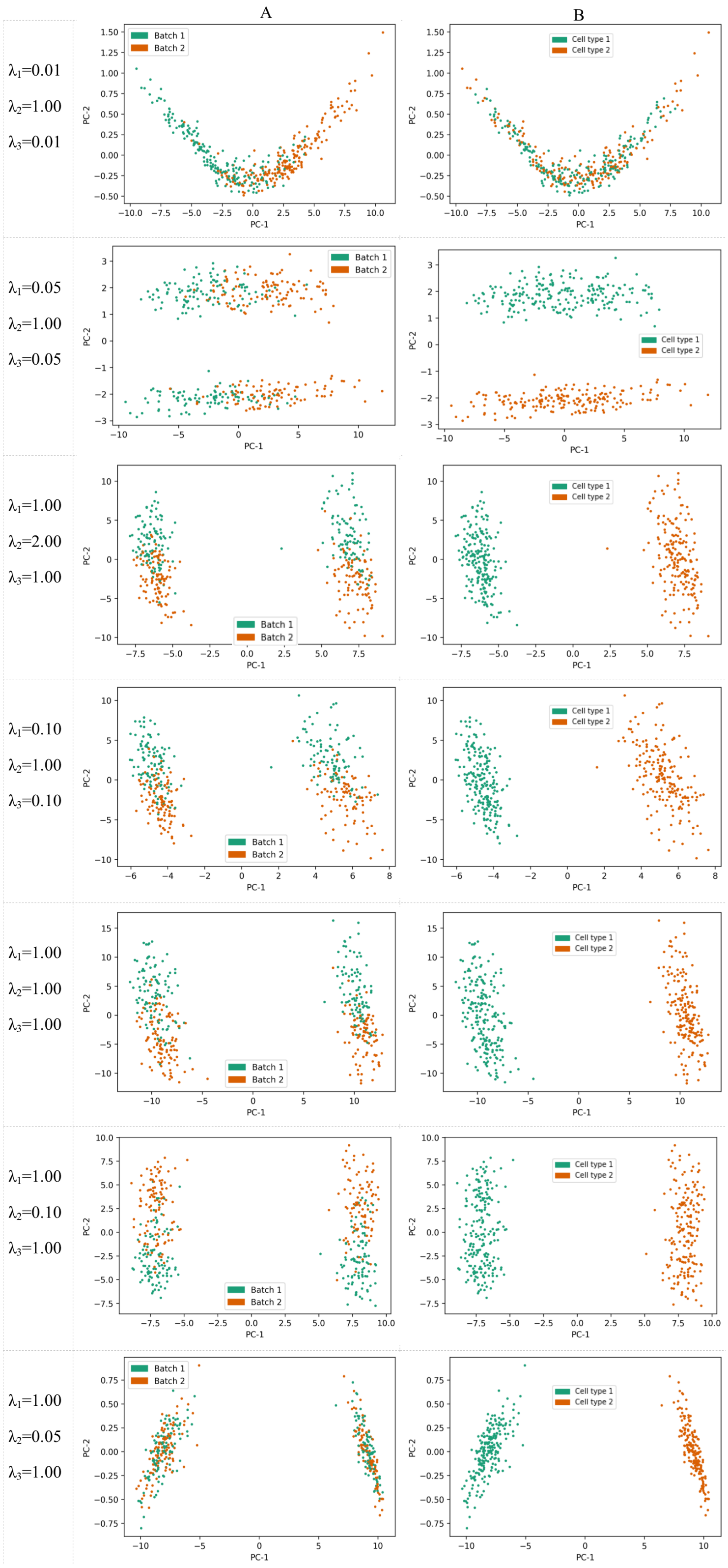

Figure S9: Impact of hyper parameters (λs) on the embeddings obtained by BEENE.(A-B) The PC plots of the embeddings learned by BEENE color coded by batches and cell types. When $\lambda_2 \ll \{\lambda_1, \lambda_3\}$, the embedding can not effectively capture batch effects. On the other hand, when $\{\lambda_1, \lambda_3\} \ll \lambda_2$, the embedding fails to capture the biological variations in the data.

2.7 Scalability to large datasets

Table S2: Time required by default kBET with PCA embedding and kBET with BEENE embedding for different datasets analyzed in this study. Default kBET was run on the whole dataset using PCA embedding. BEENE was trained on 80% of the data and kBET was run on the embedding produced by BEENE on the remaining 20% of the data (i.e., testset).

Datasets	Number of Cells	Running time (seconds)		
		kBET (whole dataset)	BEENE	
			Training	kBET (on testset)
Simulated data-1	2000	58	181	5
Simulated data- 2	1500	31	141	3
mESC dataset	5952	731	600	19
Human PMBC dataset	20886	1445	167	43
Human pancreas islet dataset	14746	451	133	25

2.8 Results of 5-fold cross validations on the simulated data

We performed a 5-fold cross-validation [8], such that circularly 80% of the data gets used as training data and the remaining 20% data is kept as test data. Therefore, dataset was divided into five equal folds. The model was trained on the four folds and tested on the remaining fold. This is repeated five times, with each fold used as the testset only once. Rejection rates and iLISI values obtained by kBET and LISI using BEENE embeddings are shown in S3.

Table S3: Results of 5-fold cross validation on the simulated datasets for different batch conditions. We show the average rejection rates and iLISI values over these five folds. The results on individual folds are also shown.

	Simulated Data with Two Cell types				Simulated Data With a Single Cell Type			
	With Two Batches		With No Batch		With Two Batches		With No Batch	
	BEENE +kBET	BEENE +LISI	BEENE +kBET	BEENE +LISI	BEENE +kBET	BEENE +LISI	BEENE +kBET	BEENE +LISI
Fold 1	0.832	1.362	0.004	1.952	0.933	1.184	0.000	1.951
Fold 2	0.854	1.266	0.014	1.955	0.875	1.312	0.075	1.935
Fold 3	0.781	1.328	0.000	1.915	0.899	1.392	0.000	1.951
Fold 4	0.667	1.411	0.065	1.948	0.807	1.342	0.118	1.918
Fold 5	0.832	1.315	0.063	1.937	0.905	1.274	0.051	1.959
Mean	0.798	1.336	0.029	1.941	0.884	1.301	0.049	1.943

3 Additional discussion

Batch effects may lead to increased spurious variability and can be confounded with real biological signal, resulting in incorrect biological conclusions. Therefore, batch effects must be assessed and accounted for in order to avoid unwanted downstream impact. However, batch effects are inherently complex and difficult to estimate. Most of the existing approaches for batch effect detection and estimation rely on a lower-dimensional embedding of the data generated by PCA. Our results on several datasets show that linear dimensionality reduction methods like PCA are not adequately sensitive to batch effects, especially in the presence of non-linearity. BEENE is a novel method, capable of generating a lower-dimensional embedding by capturing both linear and non-linear batch effects, which substantially improve the performance of existing batch estimation techniques such as kBET and LISI (compared to the performance of kBET and LISI when they use PCA embeddings).

This is both visually apparent and quantified with high accuracy reflected by appropriate iLISI values and rejection rates obtained by LISI and kBET. BEENE's improved sensitivity while retaining appropriate specificity indicates the superiority of this embedding over PCA. BEENE also demonstrated better sensitivity than PCA to the proportion of genes having batch effects. Note that BEENE–at its current form–is a batch assessment approach and hence has been compared with existing batch assessment methods in the field (eg., PCA, kBET, LISI). As a result, comparing BEENE to batch correction methods is beyond the scope of this study.

We applied BEENE to a diverse range of examples on scRNA-seq data. It was more effective than the PCA space at clustering the mESC data by batches. More importantly, the BEENE embedding was able to capture batch effects that remained after batch corrections by ComBat. On human pancreatic islet cells, the clusters based on batches and cell types were more prominent in the BEENE embedding than the PCA space. The levels of batch effects (before and after correction) were also better reflected by iLISI values and rejection rates obtained using BEENE embeddings than those using PCA embeddings. BEENE is scalable to a large number of cells because it is trained using mini batch gradient descent. On large datasets, kBET with BEENE is faster than kBET with PCA (see Supplementary Material). This is primarily due to the sample efficiency achieved by BEENE embedding, in which a portion of the entire dataset is used to run kBET and the remaining data is used to learn an embedding. Thus, methods such as kBET, which take significantly longer than other methods (e.g., LISI, which is much faster), can be made scalable to very large datasets by leveraging BEENE.

The BEENE framework lays the foundation for several future applications. This study was limited to demonstrating the feasibility and power of BEENE embedding in assessing batch effects using methods like kBET and LISI. Future studies need to extend this framework for batch correction and investigate its efficacy in batch correction methods such as Harmony and Seurat which currently rely on linear PCA embeddings. We also envision to develop a fully-automated end-to-end pipeline by leveraging the non-linear transformation of BEENE, which will not only detect but also correct for batch effects. This study demonstrates that BEENE embedding makes kBET and LISI more sensitive to the presence of linear and non-linear batch effects. Future studies need to investigate the impact of BEENE on other batch effect assessment metrics (e.g.,

ASW [9], ARI [10]). In this study we have considered the cases where the batch variables are known. However, the biological experiments can be influenced by potentially large numbers of unknown and unmeasured environmental variables [11]. As such, the BEENE framework should be enhanced to account for both known and unknown batch variables. We analyzed a large collection of data including both simulated and real scRNA-seq data. However, conducting more comprehensive experimental studies, covering a larger collection of real datasets, would be interesting to better understand and interpret the comparative performance of PCA and BEENE embeddings in batch estimation. BEENE uses a set of user defined hyper parameters (λs). Developing an improved pipeline to automatically select appropriate values of the hyper parameters is another direction to explore.

References

[1] Cosmin Lazar, Stijn Meganck, Jonatan Taminau, David Steenhoff, Alain Coletta, Colin Molter, David Y Weiss-Solís, Robin Duque, Hugues Bersini, and Ann Nowé. Batch effect removal methods for microarray gene expression data integration: a survey. *Briefings in Bioinformatics*, 14(4):469–490, 2013.

[2] Luke Zappia, Belinda Phipson, and Alicia Oshlack. Splatter: simulation of single-cell rna sequencing data. *Genome Biology*, 18(1):1–15, 2017.

[3] Maren Büttner, Zhichao Miao, F Alexander Wolf, Sarah A Teichmann, and Fabian J Theis. A test metric for assessing single-cell rna-seq batch correction. *Nature Methods*, 16(1):43–49, 2019.

[4] Hoa Thi Nhu Tran, Kok Siong Ang, Marion Chevrier, Xiaomeng Zhang, Nicole Yee Shin Lee, Michelle Goh, and Jinmiao Chen. A benchmark of batch-effect correction methods for single-cell rna sequencing data. *Genome Biology*, 21(1):1–32, 2020.

[5] Laleh Haghverdi, Aaron TL Lun, Michael D Morgan, and John C Marioni. Batch effects in single-cell rna-sequencing data are corrected by matching mutual nearest neighbors. *Nature Biotechnology*, 36(5):421–427, 2018.

[6] Tongxin Wang, Travis S Johnson, Wei Shao, Zixiao Lu, Bryan R Helm, Jie Zhang, and Kun Huang. Bermuda: a novel deep transfer learning method for single-cell rna sequencing batch correction reveals hidden high-resolution cellular subtypes. *Genome Biology*, 20(1):1–15, 2019.

[7] Laurens Van der Maaten and Geoffrey Hinton. Visualizing data using t-sne. *Journal of Machine Learning Research*, 9(11), 2008.

[8] Ron Kohavi et al. A study of cross-validation and bootstrap for accuracy estimation and model selection. In *Ijcai*, volume 14, pages 1137–1145. Montreal, Canada, 1995.

[9] Peter J Rousseeuw. Silhouettes: a graphical aid to the interpretation and validation of cluster analysis. *Journal of Computational and Applied Mathematics*, 20:53–65, 1987.

[10] Lawrence Hubert and Phipps Arabie. Comparing partitions. *Journal of Classification*, 2(1):193–218, 1985.

[11] Jeffrey T Leek and John D Storey. Capturing heterogeneity in gene expression studies by surrogate variable analysis. *PLoS Genetics*, 3(9):e161, 2007.
